# Fates of Retroviral Core Components during Unrestricted and TRIM5-Restricted Infection

**DOI:** 10.1371/journal.ppat.1003214

**Published:** 2013-03-07

**Authors:** Sebla B. Kutluay, David Perez-Caballero, Paul D. Bieniasz

**Affiliations:** 1 Aaron Diamond AIDS Research Center, Laboratory of Retrovirology, The Rockefeller University, New York, New York, United States of America; 2 Howard Hughes Medical Institute, The Rockefeller University, New York, New York, United States of America; Fred Hutchinson Cancer Research Center, United States of America

## Abstract

TRIM5 proteins can restrict retroviral infection soon after delivery of the viral core into the cytoplasm. However, the molecular mechanisms by which TRIM5α inhibits infection have been elusive, in part due to the difficulty of developing and executing biochemical assays that examine this stage of the retroviral life cycle. Prevailing models suggest that TRIM5α causes premature disassembly of retroviral capsids and/or degradation of capsids by proteasomes, but whether one of these events leads to the other is unclear. Furthermore, how TRIM5α affects the essential components of the viral core, other than capsid, is unknown. To address these questions, we devised a biochemical assay in which the fate of multiple components of retroviral cores during infection can be determined. We utilized cells that can be efficiently infected by VSV-G-pseudotyped retroviruses, and fractionated the cytosolic proteins on linear gradients following synchronized infection. The fates of capsid and integrase proteins, as well as viral genomic RNA and reverse transcription products were then monitored. We found that components of MLV and HIV-1 cores formed a large complex under non-restrictive conditions. In contrast, when MLV infection was restricted by human TRIM5α, the integrase protein and reverse transcription products were lost from infected cells, while capsid and viral RNA were both solubilized. Similarly, when HIV-1 infection was restricted by rhesus TRIM5α or owl monkey TRIMCyp, the integrase protein and reverse transcription products were lost. However, viral RNA was also lost, and high levels of preexisting soluble CA prevented the determination of whether CA was solubilized. Notably, proteasome inhibition blocked all of the aforementioned biochemical consequences of TRIM5α-mediated restriction but had no effect on its antiviral potency. Together, our results show how TRIM5α affects various retroviral core components and indicate that proteasomes are required for TRIM5α-induced core disruption but not for TRIM5α-induced restriction.

## Author Summary

The TRIM5 proteins found in primates are inhibitors of retroviral infection that act soon after delivery of the viral core into the cytoplasm. It has been difficult to elucidate how TRIM5 proteins work, because techniques that can be applied to this step of the viral life cycle are cumbersome. We developed an experimental approach in which we can monitor TRIM5-induced changes in the viral core at early times after infection, when TRIM5 exerts its effects. Specifically, we monitored the fate of the viral capsid protein, the integrase enzyme and the viral genome. We show that TRIM5 induces disassembly of each of these core components, and while some core components simply dissociate, others are degraded. These dissociation and degradation events all appear to be dependent on the activity of the proteasome. However, we also find that each of these TRIM5-induced effects events are not necessary for inhibition. The assay developed herein provides important insight into the mechanism of TRIM5α restriction and can, in principle, be applied to other important processes that occur at this point in the retrovirus life cycle.

## Introduction

Primates express a range of restriction factors that inhibit retroviral infection, and variation in restriction factors is an important determinant of retroviral tropism [1]–[3]. TRIM5α is one such factor [4], and is a member of the large family of tripartite motif (TRIM) proteins that share a common N-terminus composed of a RING domain that functions as an E3 ubiquitin ligase, one or two B-box domains required for higher-order assembly and a coiled-coil dimerization domain (RBCC) [5]–[8]. TRIM5α also encodes a variable C-terminal B30.2/SPRY domain that recognizes incoming retroviruses [4], [9]–[13] and the consequence of this recognition is that infection is inhibited soon after viral entry [14], before reverse-transcription is completed. The viral capsid (CA) protein is the direct target of TRIM5α proteins [15]–[17], and is recognized by TRIM5α multimers only in the context of assembled viral cores, but not as monomers [15], [16], [18], [19]. The RING domain of TRIM5α exhibits E3 ubiquitin ligase activity, and its removal, or mutation of key cysteine residues that are required for this activity reduces the potency of TRIM5α-mediated restriction [4], [10], [20], [21].

TRIM5α proteins with distinct spectra of antiretroviral activity are present in most, perhaps all, primate species. For example, the prototypic rhesus macaque TRIM5α (rhTRIM5α) is a potent inhibitor of HIV-1 infection but does not efficiently restrict simian immunodeficiency viruses of rhesus macaques (SIV_mac_) [4]. Human TRIM5α (huTRIM5α) and African green monkey TRIM5α (AGM TRIM5α) also exhibit antiretroviral activity [22]–[24] and although AGM TRIM5α restricts a broad range of retroviruses, huTRIM5α is known to restrict only equine infectious anemia virus (EIAV), and N-tropic MLV (N-MLV) [22]–[25]. Thus, the antiretroviral activity of TRIM5α appears to be quite plastic. Underscoring this point, in two different primate lineages (macaques and owl monkeys), independent retrotransposition events have placed a cyclophilin A (CypA) cDNA into the *TRIM5* locus, generating a fusion gene with utterly different antiretroviral specificity, wherein the B30.2/SPRY domain is replaced by CypA [26]–[29].

Although various domains of TRIM5α that are required for restriction have been well defined [7], [8], [30], the precise mechanism by which TRIM5α acts on the incoming viral cores to disrupt infection has been enigmatic. The presence of a restricting TRIM5α protein causes a decrease in the yield of pelletable CA protein following infection and, in the case of huTRIM5α restriction of N-MLV, the loss of particulate CA protein is accompanied by an increase of soluble CA [31], [32]. These experiments prompted a model whereby TRIM5α accelerates the uncoating of retroviral cores. Consistent with this model, a chimeric rhTRIM5α protein, containing the RING domain of TRIM21, lead to the shortening of capsid-nucleocapsid tubes assembled in vitro [33], [34].

A second aspect of TRIM5α-induced restriction is the role played by proteasomes. While inhibition of proteasomes does not rescue infection of restricting cells, it does rescue the formation of an integration-competent reverse-transcription complex, and appears to stabilize capsids in the cytoplasm of restricting cells [14], [25], [35]–[39]. One interpretation of these data is that TRIM5α causes a two-phase block to infection, in which passage of viral DNA to the nucleus is blocked, and then TRIM5 induces the viral core is disassembled by proteasomes. In other studies, however, inhibition of proteasomes was shown to cause a general increase in cytosolic particulate capsid independent of TRIM5 restriction [40], [41]. It has also been proposed that TRIM5α accelerates degradation of CA, by a proteasome-independent pathway [36]. In addition to acting directly on the viral core, TRIM5α has been recently shown to promote innate immune signaling, an activity that is stimulated by and may contribute to restriction of retroviral infection [42]. Overall, it is unclear what the sequence of events is during restriction, and which events are necessary or superfluous for antiviral activity.

As most studies of TRIM5-mediated restriction have focused on CA, the fate of other components of the viral core during restriction is unknown. Given that inhibition of proteasomes in restricting cells can rescue the formation of an integration competent reverse transcription complex [35], [37], one idea is that degradation of core-associated CA leads to the liberation of viral RNA and other core proteins e.g. enzymes. Thus, the physical separation of viral genomes and enzymes could lead to a block in reverse transcription. Alternatively, proteasomes may directly be involved in degradation of other core components. The lack of clarity in current pictures of how TRIM5α works is at least partly due to the difficulty of analyzing retroviral cores in infected cells using biochemical assays. This problem was partly overcome by the development of a “fate-of-capsid” assay, in which viral cores in cytosolic extracts prepared from infected cells are pelleted through a sucrose cushion [31], [32]. This approach has been utilized in a number or studies of retroviral restriction by TRIM proteins [31], [32], [39]–[41], [43]–[46] and capsid stability in infected cells [46]–[48]. Although this assay is very informative and essentially the only widely used assay for the biochemical analysis of post-entry events [31], [32], it does have limitations. First, only a fraction of the input material is actually analyzed - the endocytosed CA, which is thought to constitute the majority of the internalized material, is excluded. In addition, this approach has been applied only for the analysis of CA. Moreover, although restriction by TRIM5α likely occurs at early times after infection (i.e. 1–2 hours) [14], most studies employing this assay analyze events that take place at later stages in infection. Finally, it has been debated whether the CA analyzed during TRIM5α restriction represents viral cores in the infectious pathway [32], [36], [38], [40], as a large fraction of internalized retroviral particles are thought to be nonproductively trapped and degraded in endosomes and lysosomes [49].

In order to overcome these problems, we developed a biochemical assay by which we can monitor the effects of TRIM5α on various components of retroviral cores at early times in infected cells. The approach we took was, essentially, to elaborate existing “fate of capsid” assays. Specifically, we utilized Chinese hamster ovary K1 (CHO-K1)-derived pgsA745 cells (pgsA) which lack surface glycosaminoglycans and, perhaps as a consequence, can be very efficiently infected by VSV-G-pseudotyped viruses. Cytosolic proteins isolated from infected pgsA cells and its derivatives stably expressing various TRIM5α proteins were fractionated on linear sucrose gradients. This approach enabled the fates of CA, integrase (IN), viral genomic RNA and reverse transcription products to be monitored. Using this assay we could show that the aforementioned viral components cosediment in a dense fraction. Moreover, we found that various components of retroviral cores have different fates during TRIM5α-mediated restriction, and can be degraded or disassembled. All of these effects on retroviral cores could be at least partially blocked by proteasome inhibition, but this manipulation did not rescue infectivity. These findings suggest that events that occur prior to core disassembly, rather than core disassembly itself or the action of proteasomes, is crucial for TRIM5α-mediated restriction.

## Results

### An assay to track the fate of capsids during TRIM5α restriction

To facilitate analyses of TRIM5α-mediated restriction, we developed a biochemical assay in which we monitored various components of retroviral cores in newly infected cells. We used a CHO-derived cell line (pgsA), because it can be very efficiently infected by VSV-G pseudotyped retroviruses and does not express a TRIM5 protein that restricts MLV or HIV-1 infection [50]. Virions were bound to cells at 4°C, the inoculum was removed, and cells were either harvested immediately (T = 0 hr) or incubated at 37°C to allow infection to proceed for two hours (T = 2 hr). Our previous observations indicate that events critical for TRIM5α restriction take place during this time [14]. Extracts from infected cells were separated on linear sucrose gradients and the presence of various core components in gradient fractions assessed.

**Figure 1:**
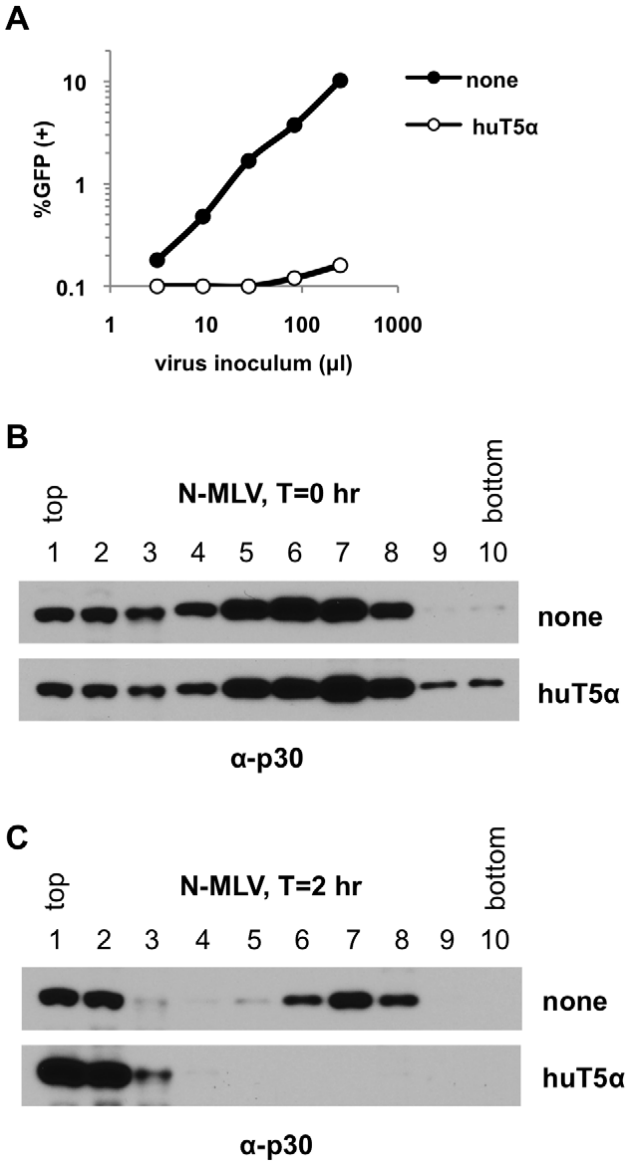
Profile of N-MLV CA isolated from pgsA and pgsA-huTRIM5α cells in sucrose gradients. PgsA cells stably expressing huTRIM5α (huT5α) and control pgsA cells (none) were infected with VSV-G pseudotyped N-MLV carrying a GFP reporter and 3 copies of HA tag in IN (IN-3×HA). (A) Infectivity of N-MLV on pgsA and pgsA-huTRIM5α cells was determined by FACS at 2 days post infection. (B, C) Cells were harvested either immediately after virion binding (T = 0 hr) or after a further incubation at 37°C for 2 hours (T = 2 hr). Post-nuclear supernatants were fractionated on 10–50% (w/v) sucrose gradients and 10 fractions from top of were collected, as explained in Materials and Methods. Western blot analysis of CA (p30) in sucrose fractions collected from T = 0 hr (B) and T = 2 hr (C) post-infection samples. Data is from a representative experiment.

Initially, we focused on N-MLV infections and characterized the effects of huTRIM5α restriction on the CA protein. As indicated in Fig. 1A, N-MLV was efficiently restricted in the pgsA-huTRIM5α cell line, as compared to unmodified pgsA cells. When cells were harvested immediately after the virion-binding step (T = 0 hr), CA was present throughout the gradient but enriched in fractions 5 to 8. This distribution is likely a consequence of virions being bound to plasma membrane fragments of varying sizes (Fig. 1B). As expected, the amount of virions bound to pgsA and pgsA-huTRIM5α cells was similar (Fig. 1B). When cells were harvested after a 2 hour incubation at 37°C following virion binding (T = 2 hr) two distinct populations of CA molecules were present in unmodified pgsA cells. One concentration of CA molecules was present at the very top of the gradient (fractions 1 and 2), and presumably represented non-particulate material. A second concentration of CA molecules that were presumably part of a larger complex was evident in fractions 6 to 8, towards the bottom of the gradient (Fig. 1C). Strikingly, the dense peak of CA protein was absent when pgsA-huTRIM5α cells were used as targets (Fig. 1C). Moreover, a clear increase of CA concentration in soluble fractions was observed (Fig. 1C). These results are consistent with prior findings using the established “fate of capsid” assay [31], [32] and imply that TRIM5α may lead to the disassembly of the capsid during the time at which TRIM5 proteins are known to exert their effects.

### Fate of the MLV integrase protein during restriction by huTRIM5α

**Figure 2:**
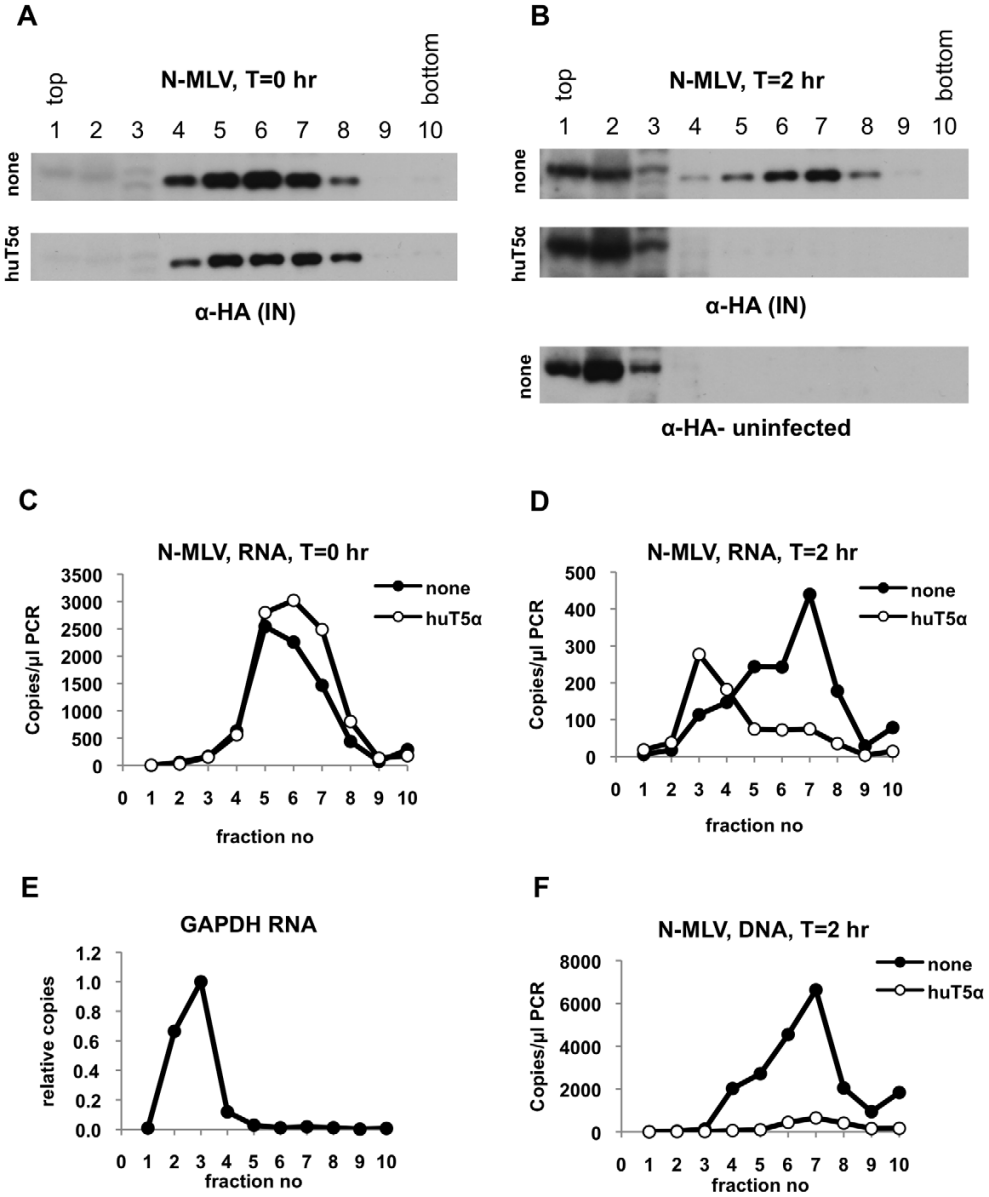
Profile of N-MLV core components isolated from pgsA and pgsA-huTRIM5α cells in sucrose gradients. PgsA cells stably expressing huTRIM5α (huT5α) and control pgsA cells (none) were infected with VSV-G pseudotyped N-MLV (IN-3×HA). Infected cells were processed as explained in legend to Fig. 1 and Materials & Methods. (A, B) Sucrose fractions collected from T = 0 hr (A) or T = 2 hr (B) samples were analyzed for the presence of integrase (IN) by western blotting using antibodies against the HA tag. (C, D) viral RNA was quantitated by Q-RT-PCR in sucrose fractions collected from T = 0 hr (C) or T = 2 hr (D) samples. (E) Q-RT-PCR analysis of cellular GAPDH RNA in fractions collected from pgsA cells. (F) Q-PCR analysis of reverse-transcription products at T = 2 hr. Data is from a representative experiment.

Although prior data [31], [32], and the findings in Fig. 1 illuminate what happens to CA protein during TRIM5α-induced restriction, the fate of other core components under restrictive conditions was unknown. Therefore, we next asked how the behavior of other components of the N-MLV cores, namely IN, viral RNA and reverse transcription products, are affected by huTRIM5α. To determine the distribution of MLV IN, we inserted a 3×HA epitope tag at its C-terminus in the context of a Gag-Pol expression plasmid. MLV particles generated using this modified Gag-Pol expression plasmid were highly infectious. Notably, when cells were harvested and subjected to analysis immediately after virion binding (T = 0 hr), IN protein was detected primarily in fractions 4 to 8 (Fig. 2A, Fig. S1A), the same fractions in which CA protein was enriched after virion binding (Fig. 1B). As expected, there was no major difference in the amount and migration pattern of IN when pgsA or pgsA-huTRIM5α cells were used. At two hours after infection (T = 2 hr) (Fig. 2B), a dense complex containing IN was detected in unmodified pgsA cells, and virtually all of the IN protein co-migrated in the gradient with the large CA containing complex identified in Fig. 1. Strikingly, the presence of huTRIM5α appeared to induce complete loss of IN at 2 h after infection (Fig. 2B). Note that a protein band detected in fractions 1–3 migrates slightly more slowly than IN and is also detected in uninfected cells (Fig. 2B) as well as when the T = 0 hr blots subjected to a longer exposure (Fig. S1A), indicating that it is a nonspecifically cross-reacting species. In contrast to the CA protein (Fig. 1C), IN was not enriched in soluble fractions under restrictive conditions (Fig. 2B), rather it appeared to be removed from cells.

### Fate of the MLV genomic RNA and reverse transcription products during restriction by huTRIM5α

We next determined the fate of viral genomic RNA during TRIM5α mediated restriction by performing quantitative RT-PCR on the gradient fractions. As was the case with the IN protein, the viral RNA was found primarily in fraction 4 to 8 of the gradient after virion binding to pgsA cells (Fig. 2C). After 2 h of infection in pgsA cells, viral RNA was found mostly in a large complex that comigrated with IN and the large CA containing complex (Fig. 2D). The co-migration of CA, IN and viral RNA suggested that they were part of the same complex, perhaps representing intact, or nearly intact viral cores. Consistent with this notion, the migration of viral RNA through the gradient (peaking at fraction 7) was very different to the migration of a cellular RNA encoding GAPDH, which was localized to fraction 3 (Fig. 2E). Notably, the presence of huTRIM5α in target cells caused a loss of viral RNA from the large complex (Fig. 2D). This huTRIM5α-induced loss of viral RNA from the large complex was accompanied by the appearance of a peak of viral RNA in fraction 3 where cellular GAPDH RNA is present (Fig. 2D, E). In other words, huTRIM5α appeared to liberate viral RNA from a sub-viral complex, causing it to adopt the behavior of a generic cellular mRNA.

Although the bulk of reverse transcription is likely not completed at T = 2 hr [14], we could easily detect reverse-transcription products in infected cells at this time point. These viral DNA species co-migrated with other components of the viral core under non-restrictive conditions (Fig. 2F). However, as expected, reverse transcription was blocked in cells expressing huTRIM5α and little viral DNA was detected anywhere on the gradient (Fig. 2F).

Given that all components analyzed that are predicted to be components of the viral core, co-fractionated with each other, it is likely that the sub-viral complexes detected herein represent functional complexes in which reverse transcription is taking place. The fact that TRIM5α clearly affected the fates of each of the viral components that were present in the dense fraction indicates that they were present in the cytoplasm, as they should not be affected by TRIM5α if they were in any other cellular location (e.g. endosomes). Moreover, these results suggest that TRIM5α-mediated restriction involves both disassembly and degradation, with the differing ultimate fates of various core components.

### Effects of huTRIM5α on MLV core components require viral entry and a restriction-sensitive CA protein

**Figure 3:**
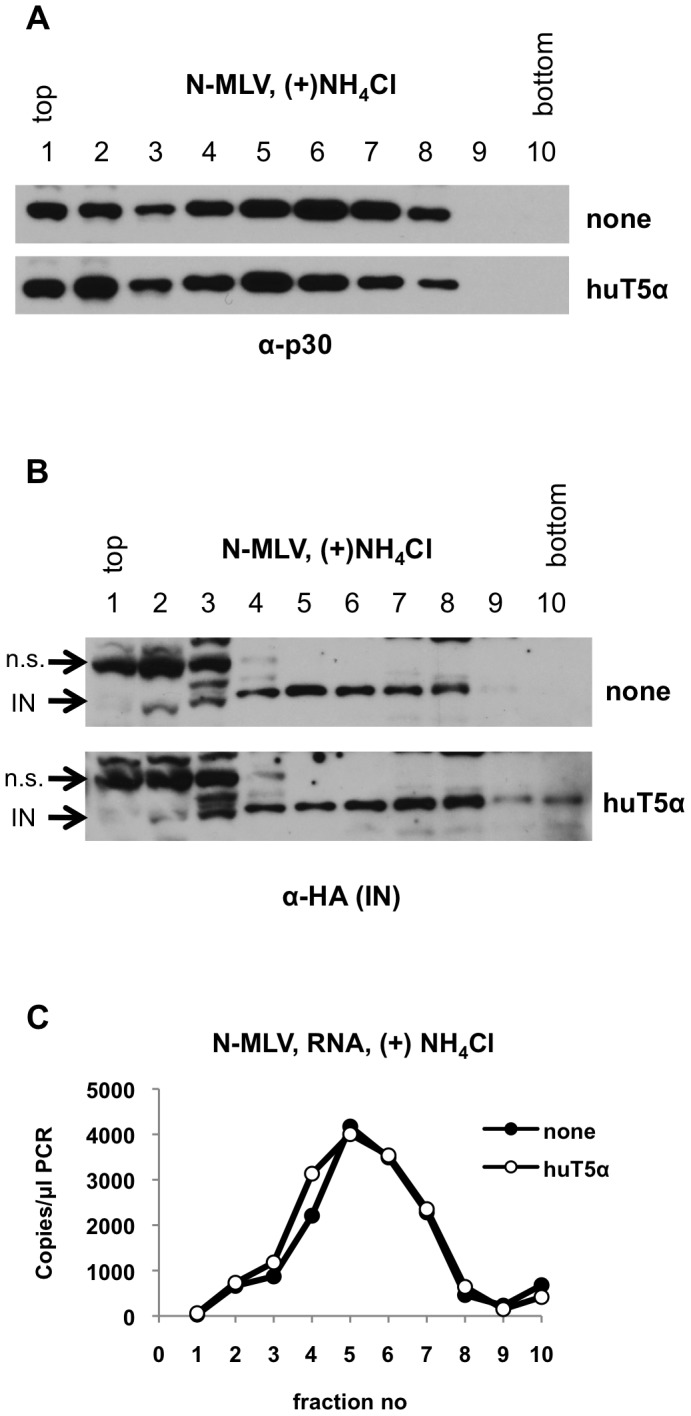
Effect of preventing endosome acidification in N-MLV infected cells. PgsA-huTRIM5α (huT5α) and control pgsA cells (none) were infected in parallel by VSV-G pseudotyped N-MLV, carrying a single HA tag in IN (IN-HA), in the presence of 50 mM NH_4_Cl for 2 hours. Samples were processed as in Fig. 1. (A, B) Proteins in sucrose fractions were analyzed by western blotting using antibodies against CA-p30 (A) and HA tag for detection of integrase (B). The non-specific (n.s.) cross-reacting protein band is indicated on the HA western blot. Note that the single HA tag-IN used in this experiments results in different migration relative to the n.s. band that appears in fractions1-3 of several of the anti-HA blots in this study. (C) Viral RNA in the same sucrose fractions was reverse transcribed and analyzed by Q-PCR. Data is from a representative experiment.

We next performed three control experiments to verify that the effects that we observed in Figs. 1 and 2 are truly relevant to restriction. Because it is thought that a significant fraction of internalized virions remain trapped in endosomes, we first asked whether the different gradient-migration patterns of core components under restrictive and nonrestrictive conditions was dependent on viral entry into the cytoplasm. To that end, cells were infected with VSV-G pseudotyped N-MLV for two hours in the presence of ammonium chloride (NH_4_Cl), which prevents endosome acidification and blocks VSV-G mediated entry. After 2 h of infection in the presence of NH_4_Cl, CA was distributed throughout the gradient (Fig. 3A), while IN (Fig. 3B) and viral RNA (Fig. 3C) were found primarily in fraction 4–8. This pattern was similar to that observed when cells were harvested immediately after virion binding, and quite different to that observed when infection was allowed to proceed for 2 h in the absence of NH_4_Cl (Fig. 1, 2). Importantly, the migration profile of the core components in the presence of NH_4_Cl was not affected by huTRIM5α. As an additional control experiment, we infected the non-restricting pgsA cells with either VSV-G-pseudotyped N- MLV (Env (+)) or N-MLV VLPs without VSV-G (Env (−)) for two hours. We could not detect CA (Fig. S2A) or IN (Fig. S2B) in the gradients prepared from cells incubated with Env (−) VLPs, This suggested that the Env(−) particles either did not efficiently bind to the target cells, or were degraded in endosomes without entering the cytoplasm. As expected, Env (−) particles were completely non-infectious (Fig. S2C) and, importantly, the initial virus inoculum contained equal amounts of Env (+) and Env (−) particles (Fig. S2D). Thus, the changes in the behavior of core components induced by huTRIM5α was dependent on VSV-G-mediated binding and entry.

**Figure 4:**
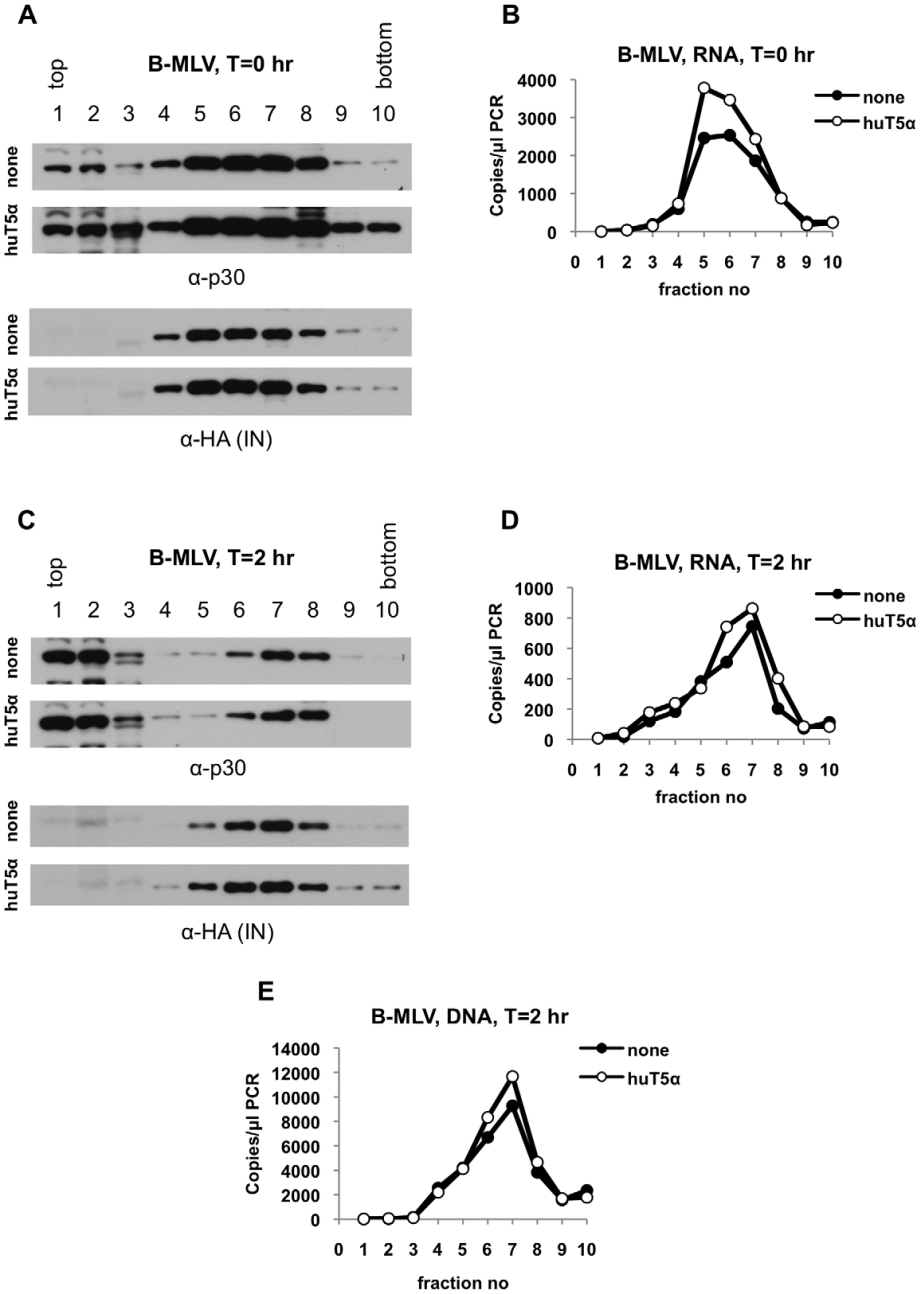
B-MLV cores isolated from both pgsA and pgsA-huTRIM5α cells migrate in dense sucrose fractions. PgsA-huTRIM5α (huT5α) and control pgsA cells (none) were infected with VSV-G pseudotyped B-MLV, carrying a GFP reporter and IN-3×HA. Infected cells were processed as explained in legend to Fig. 1 and in Materials & Methods. (A) Proteins in fractions collected at T = 0 hr were analyzed by western blotting using antibodies against CA (p30) and HA tag for detection of IN. (B) Q-RT-PCR analysis of viral RNA in fractions collected from T = 0 hr samples. (C, D) Western blot analysis of CA (p30) and IN (C) and Q-RT-PCR analysis of viral RNA (D) in fractions collected from T = 2 hr samples. (E) Reverse transcription products in fractions collected at T = 2 hr was analyzed by Q-PCR. Data is from a representative experiment.

Next, to determine whether the effects of huTRIM5α on N-MLV cores is a result of restriction activity, we performed similar experiments to those described above with B-MLV, which is insensitive to huTRIM5α restriction [22]–[25]. When viral cores were harvested after synchronization (T = 0 hr), B-MLV CA, IN (Fig. 4A, Fig. S1B) and viral RNA (Fig. 4B) co-fractionated, primarily in fractions 5 to 8, although CA was also detectable in other fractions (as was observed with N-MLV (Fig. 1–3)). All core components migrated in a similar pattern irrespective of the presence of huTRIM5α (Fig. 4A, 4B). At two hours post-infection, CA, IN (Fig. 4C, Fig. S1C) and viral RNA (Fig. 4D) were all observed as components of large complexes regardless of the presence of huTRIM5α. Moreover, unlike N-MLV, huTRIM5α did not lead to any observable increase of B-MLV CA (Fig. 4C) or viral RNA (Fig. 4D) in soluble fractions (1 to 3). As expected, the level of reverse transcription at this time point was not affected by huTRIM5α and viral cDNA co-fractionated with other core components (Fig. 4E). Collectively these results validate our assay and support the notion that changes in the behavior of viral core components are induced by a restricting TRIM5α protein.

### Proteasome inhibition restores the integrity of MLV cores under restricting conditions

The role of proteasomes during TRIM5α restriction has been a matter of debate, with several studies showing that inhibition of proteasomes does not restore infectivity that is restricted by TRIM5 proteins [14], [31], [32], [35], [37]. Moreover, in studies that employed the fate of capsid assay, huTRIM5α was reported to retain a significant ability to induce solubilization of MLV CA in the presence of proteasome inhibitor MG115 [41]. Some experiments have shown that proteasome inhibition causes a general increase in particulate HIV-1 and MLV capsids in both restricting and non-restricting cells [40], [41]. However, it has been demonstrated that proteasome inhibition can restore reverse transcription, and the formation of a functional preintegration complex in the presence of TRIM5α [35], [37]. As such, it is somewhat unclear whether proteasome inhibitor-restored reverse transcription complexes lack other core components or whether they are indistinguishable from unrestricted cores. Indeed, previous studies have indicated that viral DNA that is synthesized under TRIM5α-restricted, but proteasome inhibitor-restored conditions cannot enter the nucleus and become integrated [35], [37].

**Figure 5:**
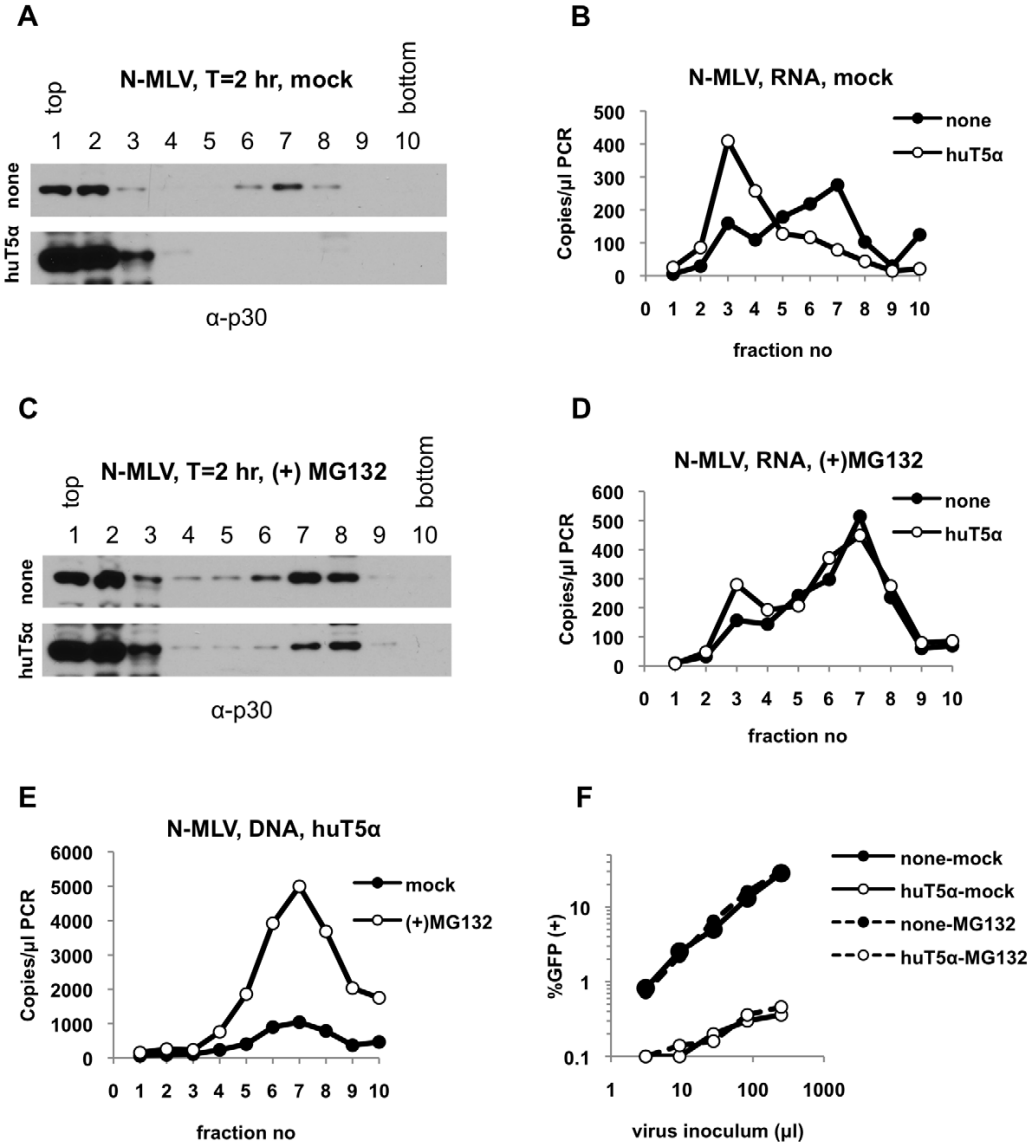
Inhibition of proteasomes in huTRIM5α expressing cells restores large subviral N-MLV complexes. PgsA-huTRIM5α (huT5α) and control pgsA cells (none) were infected with VSV-G pseudotyped N-MLV (IN-HA) in the absence (mock) or presence of 2 µM MG132 for 2 hours. Samples were processed as explained in legend to Fig. 1 and in Materials & Methods. (A, B) Protein and RNA samples isolated from mock-treated cells were analyzed by western blotting using antibodies against CA-p30 (A) and by Q-RT-PCR for detection of viral RNA (B), respectively. (C, D) CA and viral RNA in parallel fractions of MG132-treated samples were analyzed by western blotting against CA (C) and Q-RT-PCR (D), respectively. (E) Q-PCR analysis of reverse transcription products isolated from mock-treated and MG132-treated pgsA- huTRIM5α cells. (F) Infectivity of N-MLV on mock-treated and MG132-treated pgsA and pgsA- huTRIM5α cells was determined by FACS. Data is from a representative experiment.

Therefore, we asked whether inhibition of proteasomes in cells expressing huTRIM5α could restore the presence of large N-MLV sub-viral complexes containing CA, IN and viral RNA. To that end, we infected pgsA-huTRIM5α cells in the presence of MG132, a proteasome inhibitor. As observed above in Fig. 1 and Fig. 2, when huTRIM5α expressing cells were infected in the absence of MG132, large complexes containing CA (Fig. 5A) and viral RNA (Fig. 5B) were lost and there was a concomitant increase in the levels of CA and viral RNA in soluble fractions. Strikingly, MG132 treatment restored large subviral complexes containing CA (Fig. 5C) and viral RNA (Fig. 5D) as well as the formation of reverse transcription products (Fig. 5E). Importantly, in contrast to a previous study [41], we did not observe a non-specific increase in dense N-MLV capsid in cells in the presence of MG132. This may be either due to the fact that a different proteasome inhibitor was used in the study by Diaz-Griffero et al. [41] or that the indirect effects of proteasome inhibition our assays is minimized, because we analyzed an earlier time point in infection. Nonetheless, as previously reported, proteasome inhibition did not restore N-MLV infectivity in huTRIM5α cells (Fig. 5F). These results suggest that although proteasomes play an important role in mediating the observed biochemical changes on viral cores induced by TRIM5α in our assays, they are not central to TRIM5α restriction.

**Figure 6:**
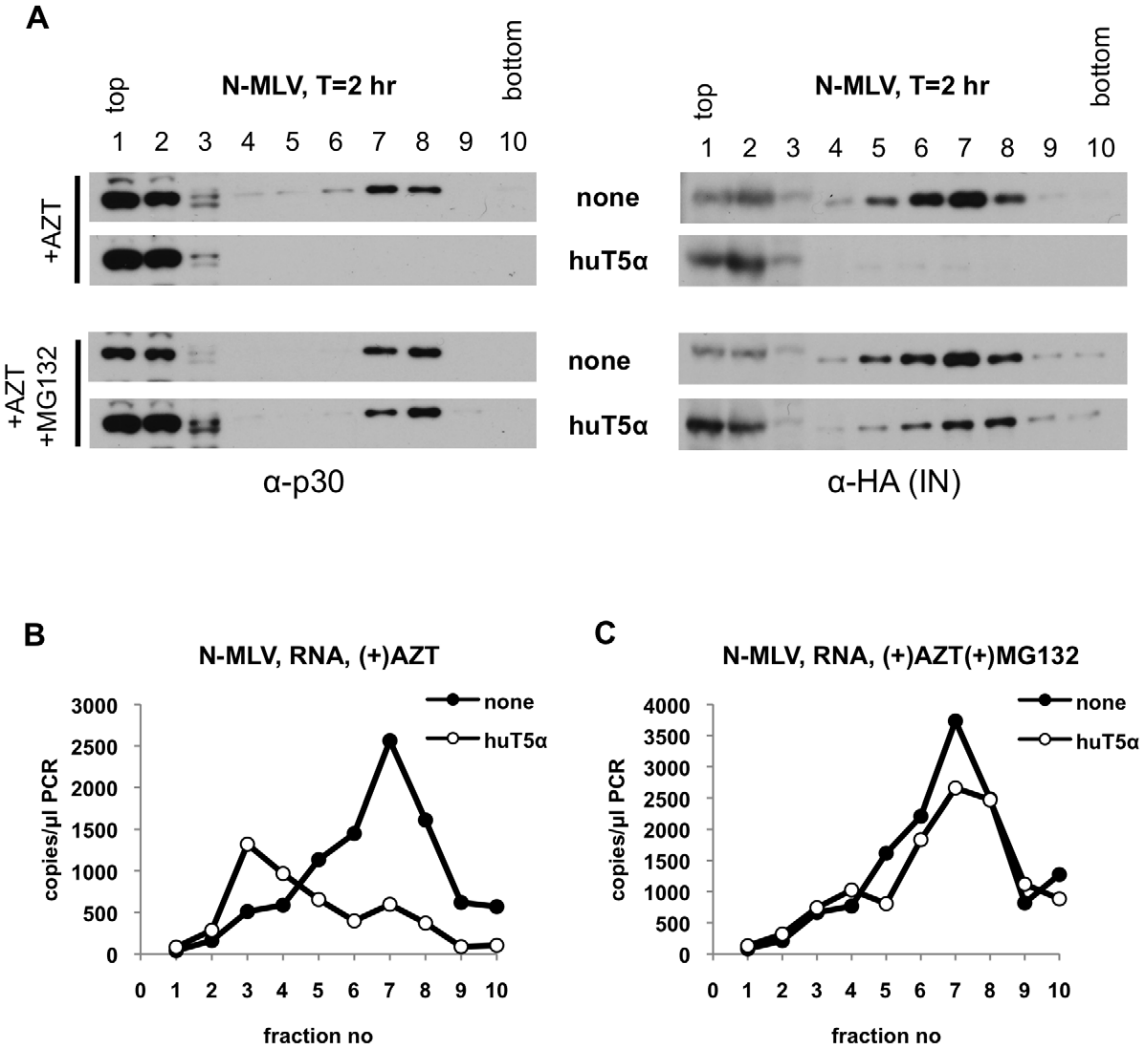
Effect of reverse transcription and proteasomes in restricting cells on large N-MLV subviral complexes. PgsA-huTRIM5α (huT5α) and control pgsA cells (none) were infected with VSV-G pseudotyped N-MLV (IN-3×HA) in the presence of either 1 mM AZT alone or 1 mM AZT together with 2 µM MG132 for 2 hours. Samples were processed as explained in legend to Fig. 1 and in Materials & Methods. (A) Protein samples in sucrose fractions were analyzed by western blotting using antibodies against CA (p30) and HA tag for detection of IN. (B, C) Viral RNA in parallel fractions of AZT treated (B) or AZT and MG132-treated (C) samples were reverse-transcribed and analyzed by Q-PCR. Data is from a representative experiment.

Recent findings have suggested the possibility that the uncoating (loss of CA protein) of HIV-1 viral cores early after infection is stimulated by reverse transcription [46], [51]. In addition, reverse transcription was suggested to be required for rhTRIM5α-mediated disassembly of the HIV-1 core using the fate of capsid assay [46]. It was possible therefore that the initiation of reverse transcription might facilitate, or even be required for, the apparent disassembly and destruction of core components that we observed. Therefore, we repeated the above experiments in the presence of the reverse transcriptase inhibitor, AZT. Importantly the doses of AZT used were sufficient to block infection (Fig. S3A), and the synthesis of reverse transcripts (Fig. S3B) under non-restricting conditions. Notably, treatment of pgsA cells with AZT during the 2 h infection assay did not affect the distribution of CA and IN in sucrose gradients: CA was present in both sets of fractions containing soluble proteins and large complexes while IN localized primarily to fractions containing large complexes (Fig. 6A). The presence of huTRIM5α in target cells led to complete disappearance of both CA and IN from large complexes, with an accompanying increase of CA in soluble fractions under these conditions (Fig. 6A). Similar to CA, even in the presence of AZT, huTRIM5α lead to the release of viral genomic RNA from the large complex (Fig. 6B). Notably, the peak of viral RNA in the presence of huTRIM5α was lower than that in its absence (Fig. 6B), suggesting that the viral RNA that is released from the core may be targeted for degradation (discussed in detail below). Inhibition of proteasomes under restricting conditions, when reverse transcription was blocked substantially restored the presence of CA, IN (Fig. 6A) and viral RNA (Fig. 6C) in large complexes. These results confirm our previous findings and suggest that huTRIM5α action involves both disassembly and proteasome-mediated degradation of viral core components, and that these events occur independently of reverse transcription.

### Effects of rhTRIM5α restriction on HIV-1 cores

**Figure 7:**
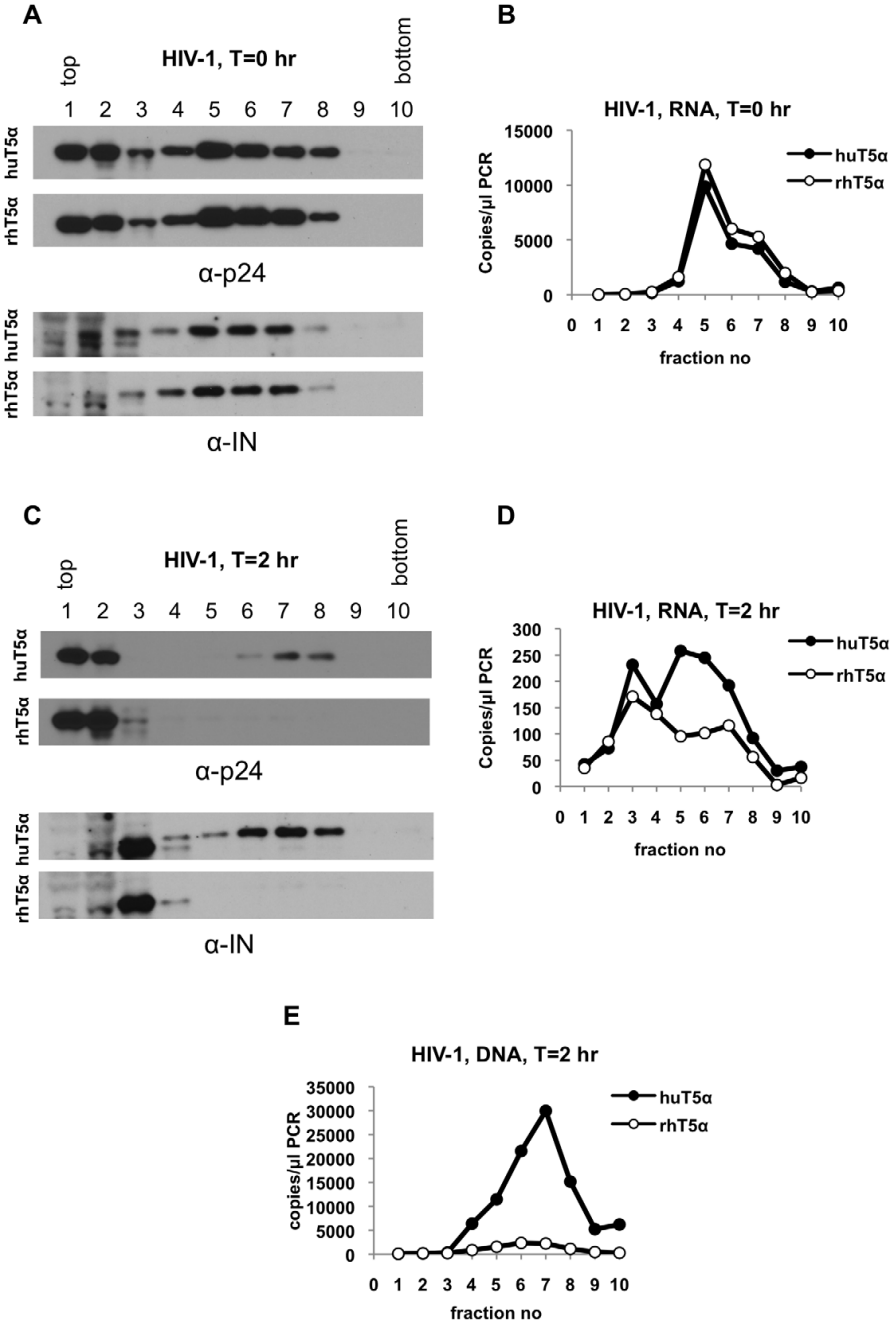
Profile of HIV-1 proteins and RNA isolated from pgsA-huTRIM5α and pgsA-rhTRIM5α cells. PgsA-huTRIM5α (huT5α) and pgsA-rhTRIM5α (rhT5α) cells were infected with VSV-G pseudotyped HIV-1, carrying a GFP reporter. Infected cells were processed at T = 0 hr and T = 2 hr as explained in legend to Fig. 1. (A) Proteins in fractions collected at T = 0 hr were analyzed by western blotting using antibodies against CA (p24) and integrase (IN). (B) Q-RT-PCR analysis of viral RNA in fractions collected from T = 0 hr samples. (C, D) Western blot analysis of CA and IN (C) and Q-RT-PCR analysis of viral RNA (D) in fractions collected from T = 2 hr samples. (E) Q-PCR analysis of viral reverse transcription products in sucrose fractions of T = 2 hr samples. Data is from a representative experiment.

We next sought to extend these observations and asked whether HIV-1 cores are similarly affected by TRIM5α restriction. To this end, we generated pgsA cells that stably express rhTRIM5α, which potently restricts HIV-1 infection (Fig. S4A). When cells expressing hu- or rhTRIM5α were harvested immediately after synchronization, CA was detected primarily in the top two fractions and in fractions 5 to 7 (Fig. 7A), whereas IN (Fig. 7A) and viral RNA (Fig. 7B) were more distinctly localized in fractions 5 to 7. As expected, there was no difference in the behavior and amounts of HIV-1 core components harvested from huTRIM5α and rhTRIM5α cells at T = 0 h (Fig. 7A, 7B). At T = 2 h post-infection, the CA protein in huTRIM5α cells was present as two distinct species with distinct migration properties in the gradient. A predominant species was present at the top of the gradient, likely corresponding to soluble proteins while a second species was present in denser sucrose fractions, likely representing viral reverse transcription complexes (Fig. 7C). The overall profile of the behavior of HIV-1 CA molecules in the sucrose gradient was quite similar to that of MLV, but the relative abundance of the soluble CA protein was greater in the case of HIV-1, suggesting the possibility that HIV-1 cores are either uncoated more rapidly following infection, or are inherently less stable in sucrose gradients. As was the case with MLV, the larger CA containing complex was lost in the presence of a restrictive TRIM5α protein (in this case rhTRIM5α, Fig. 7C). However, a corresponding increase of CA in soluble fractions was not observed, perhaps because soluble CA was already quite abundant under non-restricting conditions (Fig. 7B). Similarly, as was the case with MLV, rhTRIM5α restriction also led to the disappearance of HIV-1 IN from dense fractions, without any concurrent increase in soluble protein containing fractions (Fig. 7C).

Although rhTRIM5α restriction appeared to induce a decrease in the levels of viral RNA in dense fractions, this was not accompanied by an increase in the absolute levels of soluble RNA, although the relative amounts of soluble RNA vs. large-complex-associated RNA were increased in the presence of rhTRIM5α (Fig. 7D). This was unlike our observations with N-MLV, and makes the analysis of HIV-1 RNA profiles difficult to interpret (see discussion). Of note, under non-restricting conditions, the peak of viral RNA in dense fractions did not perfectly overlap with that of CA and IN (Fig. 7D). This could possibly be a consequence of instability of HIV-1 cores in cells or on sucrose gradients, as was suggested by the relative abundance of soluble CA versus complex-associated CA (Fig. 7C). In contrast, the products of reverse transcription co-fractionated nearly precisely with CA and IN under non-restricting condition and, as expected, were substantially reduced in rhTRIM5α cells (Fig. 7E) suggesting that the large complexes containing CA, IN and viral DNA are, or are derived from, functional HIV-1 reverse transcription complexes.

### Effects of proteasome and reverse transcriptase inhibitors on rhTRIM5α-restricted HIV-1 cores

**Figure 8:**
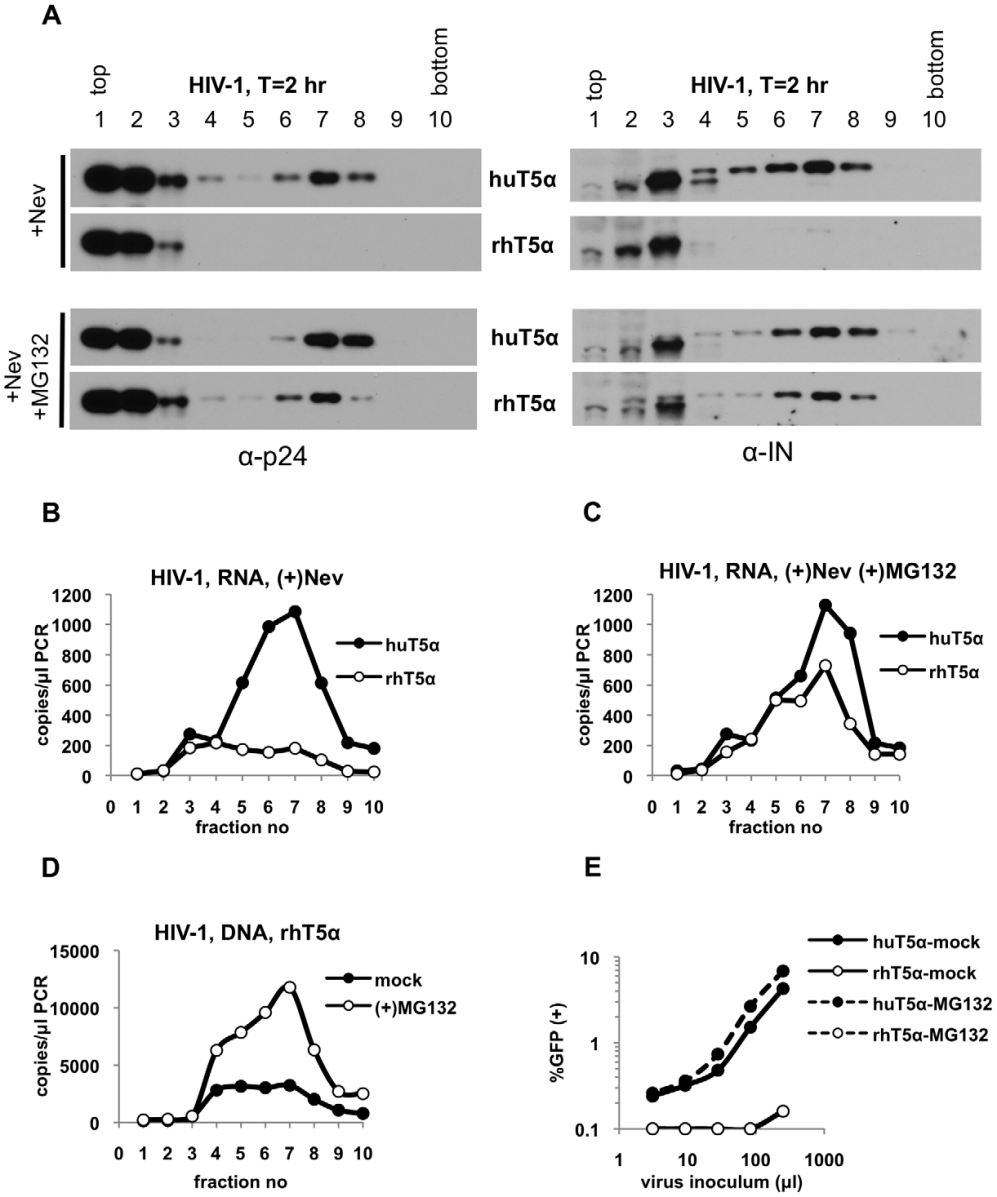
Inhibition of proteasomes restores large sub-viral complexes in HIV-1 infected pgsA-rhTRIM5a cells. PgsA-huTRIM5α (huT5α) and pgsA-rhTRIM5α (rhT5α) cells were infected with VSV-G pseudotyped HIV-1 in the presence of either 25 µM nevirapine alone or together with 2 µM MG132 for 2 hours. Samples were processed as explained in legend to Fig. 1 and in Materials & Methods. (A) Protein samples from fractions 1–10 were analyzed by western blotting using antibodies against CA (p24) and integrase (IN). (B, C) Viral RNA in parallel fractions of nevirapine-treated (B) or nevirapine and MG132-treated (C) samples was reverse-transcribed and quantitated by Q-PCR. (D, E) huT5α and rhT5α cells were infected with VSV-G pseudotyped HIV-1 in the absence (mock) or presence of 2 µM MG132 for 2 hours. Samples were processed as indicated above. (D) Q-PCR analysis of reverse transcription products isolated from mock-treated and MG132-treated pgsA-rhTRIM5α. (E) Infectivity of HIV-1 in mock-treated and MG132-treated huT5α and rhT5α cells was determined by FACS. Data is from a representative experiment.

To overcome any potential impact of reverse transcription on uncoating [46], [51], we repeated the above experiments in the presence of the reverse transcriptase inhibitor nevirapine. Importantly the doses of nevirapine used were sufficient to block infection (Fig. S4B), and reverse transcription (Fig. S4C) under non-restricting conditions. As was found with MLV, inhibition of reverse transcription in restrictive or non-restrictive cells did not affect the behavior of viral CA and IN proteins, neither of which were present in large complexes in the presence of rhTRIM5α (Fig. 8A). These results contrast with recent findings which suggest that inhibition of reverse transcription blocks the rhTRIM5α-mediated disassembly of the HIV-1 cores [46]. Notably however, nevirapine treatment of huTRIM5α cells substantially increased the level of viral RNA in dense fractions, and caused it to co-fractionate with CA and IN (Fig. 8B). This finding suggests that the poor co-fractionation of CA, IN and viral RNA observed in Fig. 7D is a consequence of reverse transcription, or RNaseH activity, rather than misbehavior of HIV-1 cores on sucrose gradients. Notably, under restricting conditions, in the presence of nevirapine, viral RNA was lost from the large complexes, with no accompanying increase in soluble fractions (Fig. 8B). This contrasts with our findings with MLV, where restriction led to an increase in the levels of soluble viral RNA.

Finally, we determined whether proteasome inhibition restored the presence of HIV-1 cores under restricting conditions. When rhTRIM5α-expressing, HIV-1 infected cells were treated with MG132 and nevirapine, CA, IN (Fig. 8A), viral RNA (Fig. 8C) and reverse transcription products (Fig. 8D) were all significantly restored in dense fractions. However, as was the case with restricted MLV infection, proteasome inhibition did not restore virus infectivity (Fig. 8E). It is important to note that, unlike a previous study [40], we did not observe a non-specific increase in particulate capsid in cells in the presence of MG132. This may be either due to the fact that our assays are performed at much earlier time points post-infection, which may minimize indirect effects of proteasome inhibition, or that a different proteasome inhibitor was used in the study by Diaz-Griffero et al. [40]. Nonetheless, these results suggest that rhTRIM5α modifies the HIV-1 cores in a way that likely leads to the degradation of both IN and viral RNA. Although this process is sensitive to proteasome inhibition, but is not required for the antiviral activity of TRIM5α to be manifested.

### Effects of proteasome and reverse transcriptase inhibitors on omkTRIMCyp-restricted HIV-1 cores

**Figure 9:**
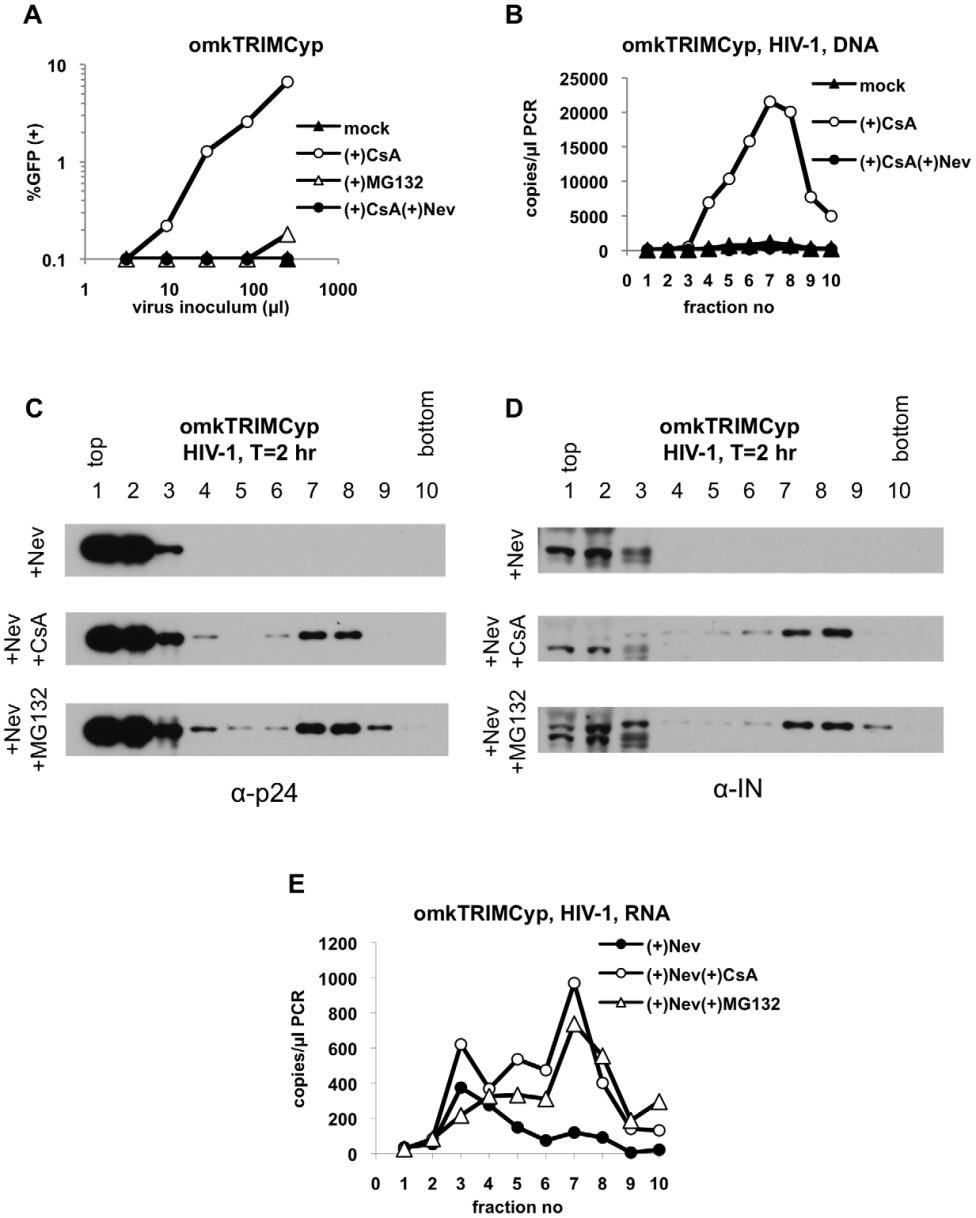
Inhibition of proteasomes restores large sub-viral complexes in HIV-1 infected omkTRIMCyp-expressing cells. (A) Infectivity of HIV-1 in mock-, cyclosporin A (CsA, 5 µM)-, MG132 (2 µM)-, and CsA and nevirapine (25 µM)-treated pgsA-omkTRIMCyp cells was determined by FACS. (B) pgsA-TRIMCyp cells were infected with VSV-G pseudotyped HIV-1 in the absence (mock) or the presence of 5 µM CsA alone, or together with 25 µM nevirapine. Samples were processed and reverse transcription products were analyzed by Q-PCR as explained above. (C–E) PgsA-omkTRIMCyp cells were infected with VSV-G pseudotyped HIV-1 in the presence of either 25 µM nevirapine alone or together with 5 µM cyclosporine A (CsA) or 2 µM MG132 for 2 hours. Samples were processed as explained in legend to Fig. 1 and in Materials & Methods. Protein samples from fractions 1–10 were analyzed by western blotting using antibodies against CA (p24, C) and integrase (IN, D). Viral RNA in parallel fractions were reverse-transcribed and analyzed by Q-PCR (E). Data is from a representative experiment.

We then sought to confirm there observations by asking whether similar changes on HIV-1 cores can be induced by a different restrictive TRIM protein, namely owl monkey TRIMCyp (omkTRIMCyp) [27], which was shown previously to reduce the amount of pelletable capsid in a fate of capsid assay [32], [40], [43]. This experimental system is better internally controlled, as restriction by omkTRIMCyp protein can be overcome by treatment of cells by cyclosporin A (CsA), which prevents its binding to viral CA protein [27]. Since restricted and non-restricted HIV-1 core components were more reliably compared in the presence of reverse transcriptase inhibitor nevirapine (Fig. 8), we performed similar experiments in pgsA-omkTRIMCyp cells in its presence. As expected, although the treatment of pgsA-omkTRIMCyp cells with CsA restored infectivity (Fig. 9A) and reverse transcription (Fig. 9B), the doses of nevirapine used in these experiments were sufficient to block both of these processes (Fig. 9A, B). In omkTRIMCyp-expressing cells treated with nevirapine alone, viral CA (Fig. 9C), IN (Fig. 9D) and viral RNA (Fig. 9E) were absent in dense fractions, without any notable increase in soluble fractions, similar to our observations with rhTRIM5α. As expected, large sub-viral complexes containing CA (Fig. 9C), IN (Fig. 9D) and viral RNA (Fig. 9E) were restored in the presence of CsA. Importantly, when pgsA-omkTRIMCyp cells were treated with MG132 and nevirapine, CA (Fig. 9C), IN (Fig. 9D) and viral genomic RNA (Fig. 9E) were all restored in dense fractions to almost the same level as observed under CsA treatment. However, as it was the case with restricted MLV and HIV-1 infections, proteasome inhibition did not restore virus infectivity (Fig. 9A). These results together show that omkTRIMCyp disrupts HIV-1 cores in a similar way to that of rhTRIM5α and leads to degradation of at least some core components. Likewise, although this process is sensitive to proteasome inhibition, proteasomes are not required for the antiviral activity of omkTRIMCyp.

## Discussion

We formulated an experimental approach in which the fates of multiple viral core components can be tracked in infected cells, with the aim of understanding how TRIM5α restricts retroviral infection. The approach is similar in principle to the “fate of capsid” assay [31], [32], in which the putative separation of viral cores from infected cell lysates on sucrose gradients enables the analysis of their composition. However, our assay is more elaborate, and perhaps more effective, in several aspects. First, we monitored TRIM5α- and TRIMCyp-induced changes not only for CA, but also for IN, viral RNA and reverse transcription products in the same fractionation experiment. Second, in our assay, all of the input cellular material is analyzed, without the need for exclusion of putatively endocytosed virions. Although it is generally held that the majority of retroviral particles become trapped in endosomes of target cells, complicating analysis of early events in infection, this did not seem to be a major problem in our experiments. Indeed, the nearly complete disappearance of IN at T = 2 h specifically from restricting cells, argues that there is very little virus associated with the cells that had not reached the cytoplasm by this time point. Although the reasons for this are not clear, possibilities include highly efficient VSV-G-mediated entry in pgsA cells, particular instability of endocytosed virions in pgsA cells, or the fairly low MOIs used in these experiments. Third, infections are fully synchronized and the unbound input virus is removed before infection, which could limit the number of virions that are nonspecifically endocytosed. Fourth, analysis is carried out at an early time (2 h) after infection when events relevant to TRIM5 restriction occur [14]. Fifth, we have incorporated quantitative aspects in our experimental system: Q-PCR analysis of viral RNA has proven to be an accurate and quantitative indicator of the fate of the viral core undergoing TRIM5α restriction. Overall our findings suggest that of all of the above components are present in a large complex comprising all or part of the virion core that is a functional intermediate in the infection pathway.

**Figure 10:**
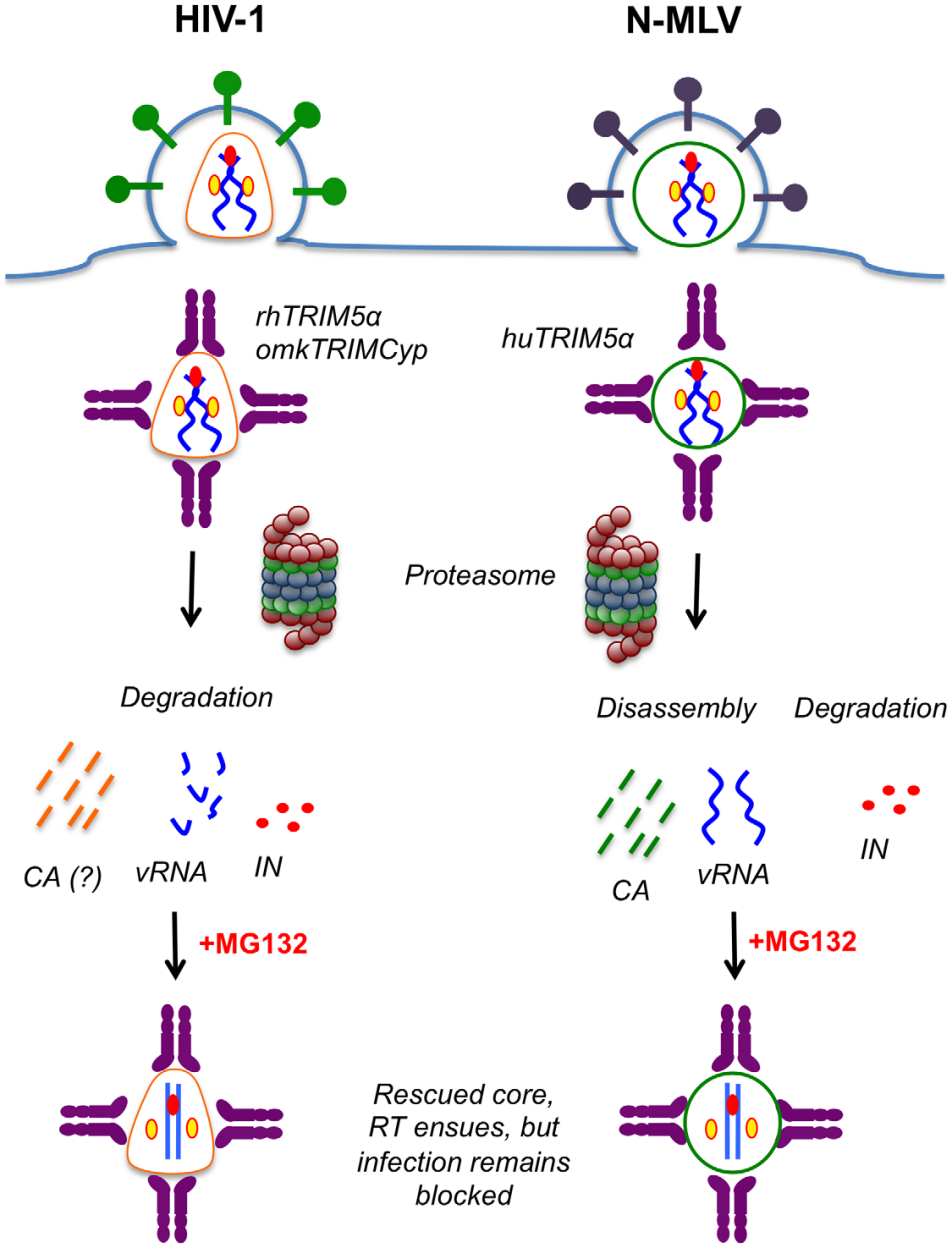
Model for mechanism of restriction by TRIM5 proteins. Soon after entry into the cytoplasm, retroviral cores are recognized by TRIM5 proteins, which leads to both disassembly and degradation of core components. Inhibition of proteasomes prevents both of these events and restores viral cores where reverse transcription can take place. However, inhibition of proteasomes does not restore virus infectivity indicating that another event (e.g. coating of viral cores by TRIM5 proteins) is crucial for restriction.

Our findings provide insight into events that take place during TRIM5α restriction (Fig. 10). In parallel with previous findings [31], [32], we observed that N-MLV CA was redistributed from large complexes to soluble fractions in cells expressing huTRIM5α (Fig. 1C). We expanded these observations and show that viral RNA was released from large complexes as a result of huTRIM5α restriction (Fig. 2D). In contrast, MLV IN was not retained in a soluble form following its loss from dense fractions, and appeared to be degraded (Fig. 2B, Fig. 10).

HIV-1 differed from MLV in that neither HIV-1 CA nor viral RNA was apparently increased in soluble fractions concurrent with their loss from large complexes (Fig. 7B, 7D, 8C, 8E). However, the comparative pre-existing abundance of CA in soluble fractions may have masked any redistribution of CA protein to those fractions. Possible reasons for the discrepant fate of MLV and HIV-1 RNA under restricting conditions are discussed below. In the case of HIV-1 IN, the protein was lost from cells under restricting conditions in much the same way as was observed for MLV. Collectively these results indicate that TRIM5α causes both disassembly and degradation of viral components with similarities and differences in the fates of individual core components across retroviral genera (Fig. 10).

Recent findings have suggested the possibility that the uncoating of retroviral cores early after infection is stimulated by reverse transcription [46], [51] and that rhTRIM5α-mediated disassembly of HIV-1 cores requires reverse transcription activity [46]. Although in some experiments reverse transcriptase inhibitors modestly increased the amount of capsid detected by western blotting, we did not observe any effect of RT inhibitors on TRIM5-mediated disassembly/degradation of cores in this study. The reasons underlying the discrepancy between our results and the study by Yang et al. [46] are not clear. However, one would predict that reverse transcription is not required for restriction by TRIM5, based on the fact that TRIM5 acts rapidly after entry [14], before majority of reverse transcription is completed.

The precise role of proteasomes in TRIM5-mediated restriction has been difficult to unambiguously determine. As previously demonstrated [35], [37], inhibition of proteasomes in restricting cells restored MLV and HIV-1 reverse transcription (Fig. 5E, 8D). Importantly, we found that proteasome inhibition restored a core complex that is biochemically indistinguishable from unrestricted viral cores, and contained CA, IN and viral RNA (Fig. 5, 6, 8). As such, it is unlikely that TRIM5α mediates the complete disassembly of cores without the aid of proteasomes. Nevertheless, it is clear that proteasomes are not required for restriction by TRIM5α, as MG132 treatment of restricting cells does not restore virus infectivity ([14], [31], [32], [35], [37] and Fig. 5F, 8E, 9A). Recent findings suggest that TRIM21/TRIM5α chimeras have the propensity to form hexameric lattices on the HIV-1 core, and it is possible that this phenomenon, in itself, constitutes the underlying mechanistic basis for restriction [52]. The assembly of such a lattice on the core may block the targeting of viral reverse-transcription or pre-integration complexes to the nucleus, because circular viral DNA forms are not generated during restricted HIV-1 infection under conditions of proteasome inhibition [35], [37]. However, because HIV-1 and MLV apparently have different underlying mechanisms of entering the nucleus, it is possible that the other mechanisms that sequester viral DNA (e.g. failure to uncoat) may underlie the inability of HIV-1 or MLV to productively infect restrictive cells under conditions of proteasome inhibition.

It is intriguing that some N-MLV and HIV-1 core components, notably viral RNA (and perhaps CA), have somewhat different fates under restrictive conditions (Fig. 10). A possible explanation for this difference is that N-MLV core components are intrinsically more stable and as such, are degraded at a slower rate after TRIM5α-induced disassembly. Alternatively, rhTRIM5α and omkTRIMCyp may either specifically recruit a cofactor that more efficiently degrades the core components or simply disassemble HIV-1 cores at a faster rate. The loss of both N-MLV and HIV-1 IN in dense fractions without any apparent increase in soluble fractions may reflect the previously reported intrinsic instability of these proteins [53].

We did not detect obvious ubiquitinylation of any core proteins undergoing restriction in our assays. It is conceivable that ubiquitin-independent degradation or disassembly by proteasomes may be important for the observed effects on the cores [54]–[59]. Alternatively, if TRIM5 is responsible for ubiquitin modification of only a small fraction of core-associated proteins (e.g. CA), we would not be able to detect this modification yet it could be responsible for core disassembly.

The most striking difference between HIV-1 and MLV restriction is the fate of the viral RNA following its release from the core. It appears that MLV RNA is largely preserved, in a soluble form, whereas HIV-1 RNA is lost. We speculate that the mechanism by which HIV-1 viral RNA is lost during restriction is related to its nucleotide composition. It has long been known that the high AU content destabilizes the HIV-1 genome [60]–[66]. It is therefore conceivable that once the HIV-1 genome is exposed in the cytosol as a result of restriction, AU-rich elements may lead to the degradation of the genome, in the same way as has been observed with several RNAs coding for oncoproteins and growth factors [67]. Alternatively, proteasomes themselves, which have been suggested to comprise RNase activity, or other putative TRIM5α associated RNase activities may lead to selective degradation of AU-rich viral RNA molecules [68]. Nevertheless, it is unlikely that RNA degradation is critical for TRIM5 restriction as TRIMCypA chimeras containing the RBCC domain from other TRIM proteins, certain RING domain mutants of TRIM5α and squirrel monkey TRIM5α can restrict HIV-1 and SIV_mac_ infection, respectively, after reverse transcription is completed [39], [69], [70].

TRIM5α mediated restriction serves as a useful model on which to base investigations of post-entry events. As such, the assay developed here could also be utilized to study restriction-independent events in newly infected cells. For example, it has been suggested that retroviral cores are optimally stable, and changes in CA stability in vitro can lead to defects in reverse transcription [71]. The assay developed here could identify the effects of such changes on multiple viral core components in infected cells. However, a caveat of our assay is that the precise nature of the ‘large complexes’ to which we refer is not known. For instance, it is not known whether the large complexes containing CA and the cofractionating core components actually represent intact conical viral cores. Previous investigations of cores isolated from extracellular virions and infected cells revealed notable differences in the density of N-MLV, B-MLV and HIV-1 ‘cores’ [31], [71]–[74]. We did not observe such differences in our assays, as separation of cytosolic extracts in our experimental system is based on size, rather than density. Therefore, it is plausible that MLV and HIV-1 cores of different densities migrate almost identically on the sucrose gradients as they have similar sizes. Notably, even under non-restrictive conditions, a significant fraction of CA is present in soluble fractions. A similar phenomenon has been previously observed by others during isolation and biochemical characterization of HIV-1, and to a lesser extent MLV, reverse transcription complexes in infected cells [75], [76] This could be a consequence of the disassembly of some fraction of CA immediately upon infection or of the fact that only a proportion of the virion CA protein is actually assembled into cores in mature virions [77]. It is unlikely that the soluble CA represents complete disintegration of a fraction of viral cores in dense sucrose gradients [71], [73], as neither viral RNA nor IN is solubilized under non-restrictive conditions.

Overall, we devised a novel experimental approach in which events that take place during TRIM5α restriction can be analyzed, and that can be applied generically to the study of early events in retrovirus replication cycle. Our results indicate that viral core components have distinct fates during TRIM5α restriction and are either disassembled or degraded. Importantly, in line with the two-step mechanism previously proposed [35], [37], [39], [69], although the TRIM5α-induced biochemical changes on the viral cores in our assays are sensitive to proteasome inhibition, proteasomal degradation is clearly not required for restriction. Future studies will address by which mechanism TRIM5α can restrict retrovirus infection as well as the mechanistic details of how different core components are affected by restriction.

## Materials and Methods

### Cells and viruses

CHO K1-derived pgsA-745 cells (CRL-2242, ATCC) and all of its derivatives were maintained in Ham's F12 media (Life technologies, 11765-054) supplemented with 10% fetal bovine serum and 1 mM L-glutamine. HEK 293T cells were obtained from ATCC (CRL-11268) and maintained in Dulbecco's modified Eagle's medium supplemented with 10% fetal bovine serum. VSV-G pseudotyped viruses were produced by transfection of 293T cells with plasmids expressing HIV-1 or MLV Gag-Pol, a packagable vector genome (see below) carrying GFP [15], [78] and VSV-G at a ratio of 5∶5∶1, respectively, using polyethyleneimine (PolySciences, Warrington, Pennsylvania, United States) as described previously [79].

### Plasmids

Sequences encoding huTRIM5α, rhTRIM5α and omkTRIMCyp were inserted into LNCX retroviral vector plasmid (Clontech), which were subsequently used to generate cloned pgsA-745 cell lines stably expressing huTRIM5α and rhTRIM5α. MLV and HIV-1 vector genome plasmids, CNCG and CCGW, respectively, encode a GFP reporter under the control of CMV promoter [15], [78]. NL4-3 derived HIV-1 Gag-Pol sequence were inserted into the pCRV-1 plasmid [80] and carry a hemagglutinin (HA) tag at the C-terminus of integrase (pNL-GP IN-HA). Sequences encoding B-MLV and N-MLV Gag-Pol inserts carrying a single copy or three copies of HA-tag at the C-terminus of integrase were inserted into pCAGGS plasmid [81]. Further details of plasmids are available upon request.

### Infections and restriction assays

PgsA745 cells, or derivatives thereof, (4×10^6^) were plated on 10-cm cell culture dishes one-day before infection. For each treatment and time point, two such 10-cm dishes were used. In parallel, 2.5×10^4^ PgsA745 cells were plated in 24-well plates to determine virus infectivity in each experiment. The corresponding MOI on 10-cm dishes was ∼0.025 for MLV infections and ∼0.01 for HIV-1 infections. Cell culture supernatants containing VSV-G pseudotyped viruses were filtered and treated with RNase free DNaseI (Roche) at a concentration of 1 unit/ml for 1 hour at 37°C in the presence of 6 mM MgCl_2_. Cells were washed with ice-cold phosphate-buffered saline (PBS) and 6–7 ml of chilled virus (adjusted to contain 20 mM HEPES) was added to the cells. After allowing virus binding to cells at 4°C for 30 minutes, the inoculum was removed and cells were washed three times with PBS. Parallel infections were carried to determine the infectious titer in a given experiment. Cells were either harvested immediately (T = 0 hr) or incubated at 37°C for 2 hours (T = 2 hr) in complete cell culture media. In some experiments, cyclosporine A, proteasome and reverse transcriptase inhibitors were included during virion binding and during incubation at 37°C. Cells were collected in 1X PBS-EDTA, pelleted and resuspended in 1 ml of hypotonic buffer (10 mM Tris-Cl pH 8.0, 10 mM KCl, 1 mM EDTA supplemented with complete protease inhibitors (Roche) and SuperaseIN (Life technologies)). After incubation on ice for 15 minutes, cell suspension was dounce homogenized by 50 strokes, using pestle B. The disruption of cells and the integrity of nuclei were monitored by Trypan blue staining of cells and nuclei (Fig. S5). Nuclear material was pelleted by centrifugation at 1000×*g* for 5 minutes and post-nuclear supernatant was layered on top of a 10–50% (w/v) linear sucrose gradient prepared in 1X STE buffer (100 mM NaCl, 10 mM Tris-Cl (pH 8.0), 1 mM EDTA). Samples were ultracentrifuged on a SW50.1 rotor at 30000 rpm for 1 hour. Ten 500 µl fractions from top of the gradient were collected, and proteins, RNA and DNA in each fraction was analyzed as described below.

### Western blotting

Proteins in each sucrose fraction were precipitated by trichloroacetic acid as described previously [82]. Protein pellets were resuspended in 50 µl of 1X protein sample buffer and analyzed by western blotting. The primary antibodies used were: mouse monoclonal anti-HA (HA.11 Covance), mouse monoclonal anti-HIV-1 p24CA (183-H12-5C NIH), mouse monoclonal anti-HIV-1 IN (a gift from Michael Malim) and goat polyclonal anti-MLV p30 (a gift from Stephen Goff).

### Quantitation of viral RNA and DNA

For analysis of DNA and RNA, 50 µl of each fraction of the sucrose gradient was digested with proteinase K, phenol∶chloroform extracted and precipitated using sodium acetate/ethanol as described previously [82]. For analysis of RNA, samples were further treated with DNase I, extracted again and reverse-transcribed using the ImProm-II reverse transcription kit (Promega). The resulting cDNA and DNA samples were used as template for quantitative real-time PCR (qPCR) using FastStart Universal SYBR Green Master Mix (Roche) and ABI 7500 Fast PCR system. PCR primers were designed within the GFP region of the vector genome. The primer pairs used in this study are as follows: GFP: Forward: *5′ AAGTTCATCTGCACCACCGGCAA* Reverse: 5′ *TGCACGCCGTAGGTCAGG* ; GAPDH: Forward: *5′ AGG TGA AGG TCG GAG TCA ACG*, Reverse: *5′ GGT CAT TGA TGG CAA CAA TAT CCA CTT TAC*.

## Supporting Information

Figure S1 **Longer exposures of IN-HA western blots in this study.** (A) Longer exposure of the western blots in Fig. 2A. (B) Longer exposure of the western blots in Fig. 4A. (C) Longer exposure of a western blot from repetition of an experiment performed in Fig. 4C.
(TIF)

Figure S2 **Effect of preventing viral entry in N-MLV infected pgsA cells.** PgsA cells were infected for 2 hours as above with either a VSV-G-pseudotyped virus (Env (+)) or N-MLV VLPs lacking VSV-G (Env (−)). Cells were processed and analyzed on gradients as normal. (A) Western blot analysis of CA (p30) in gradient fractions (B) Western blot analysis of IN in gradient fractions using an antibody against the HA-tag. (C) Infectivity of N-MLV Env(+) and N-MLV Env(−) on pgsA cells was determined by FACS at 2 days post infection. (D) The input virus inoculum was pelleted through 20% sucrose and analyzed by western blotting using antibodies against CA (p30) and IN.
(TIF)

Figure S3 **AZT blocks N-MLV infection and reverse transcription.** (A, B) PgsA cells were infected with VSV-G pseudotyped N-MLV (IN-3×HA) either in the absence (mock) or in the presence of either 1 mM AZT as explained in legend to Fig. 1 and in Materials & Methods. (A) Cells were fixed and virus infectivity was determined by FACS at 2 days post infection. (B) Q-PCR analysis of reverse transcription products isolated from mock-treated and AZT-treated pgsA cells.
(TIF)

Figure S4 **RhTRIM5α and Nevirapine block HIV-1 infection and reverse transcription.** (A) PgsA-huTRIM5α (huT5α) and pgsA-rhTRIM5α (rhT5α) cells were infected with VSV-G pseudotyped HIV-1 as above and infectious titer was determined by FACS at 2 days post infection. (B, C) PgsA-huTRIM5α cells were infected by VSV-G pseudotyped HIV-1 either in the absence (mock) or in the presence of either 25 µM nevirapine as explained in legend to Fig. 1 and in Materials & Methods. (B) Cells were fixed and virus infectivity was determined by FACS at 2 days post infection. (C) Q-PCR analysis of reverse transcription products isolated from mock-treated and AZT-treated pgsA cells at 2 h post infection.
(TIF)

Figure S5 **Dounce homogenization of pgsA cells.** PgsA cells that were processed the same way as for the infected samples were dounce homogenized in hypotonic buffer as explained in Materials & Methods. Cell integrity before homogenization (in hypotonic buffer) and after 50 strokes was determined by Trypan blue staining of cells, and using a Countess automated cell counter. Integrity of nuclei after 50 strokes was determined by counting Trypan blue stained nuclei. Data is presented as the percentage of the initial number of cells.
(TIF)
