# Supplementary figures and images for: Fates of Retroviral Core Components during Unrestricted and TRIM5-Restricted Infection
Source: PLoS Pathog. 2013 Mar 7;9(3):e1003214. doi: 10.1371/journal.ppat.1003214 (PMC3591316; doi:10.1371/journal.ppat.1003214)

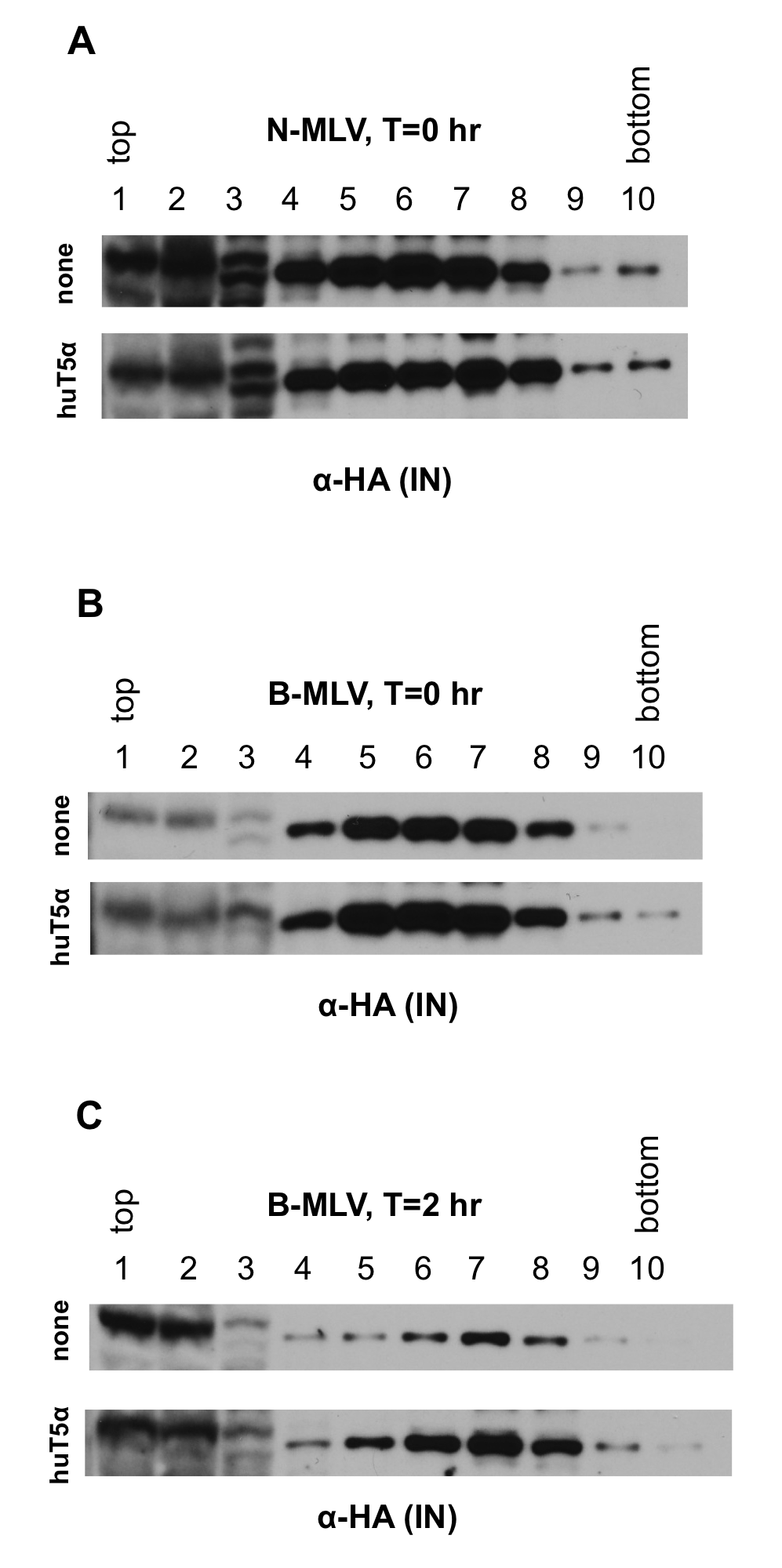

Supplement: Figure S1 — Longer exposures of IN-HA western blots in this study. (A) Longer exposure of the western blots in Fig. 2A. (B) Longer exposure of the western blots in Fig. 4A. (C) Longer exposure of a western blot from repetition of an experiment performed in Fig. 4C. (TIF) [file ppat.1003214.s001.tif]

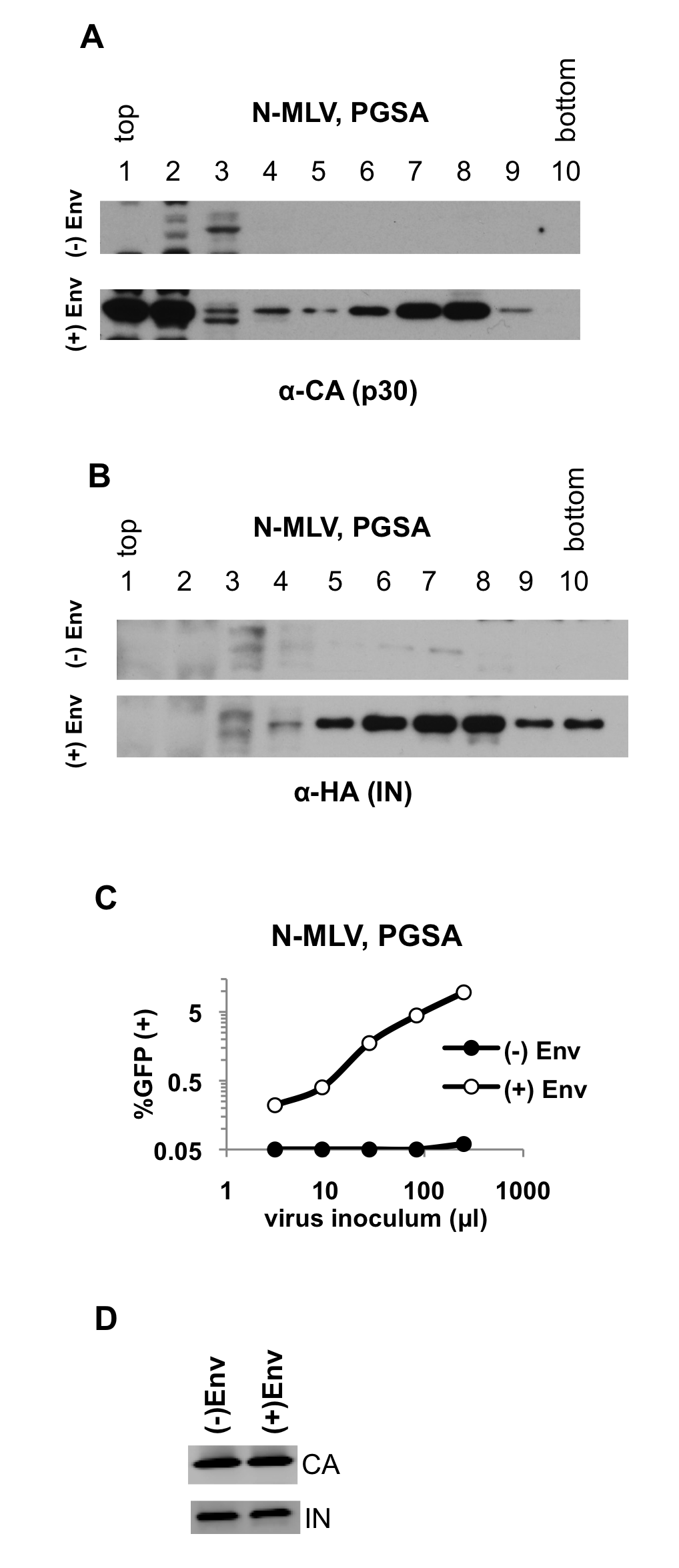

Supplement: Figure S2 — Effect of preventing viral entry in N-MLV infected pgsA cells. PgsA cells were infected for 2 hours as above with either a VSV-G-pseudotyped virus (Env (+)) or N-MLV VLPs lacking VSV-G (Env (−)). Cells were processed and analyzed on gradients as normal. (A) Western blot analysis of CA (p30) in gradient fractions (B) Western blot analysis of IN in gradient fractions using an antibody against the HA-tag. (C) Infectivity of N-MLV Env(+) and N-MLV Env(−) on pgsA cells was determined by FACS at 2 days post infection. (D) The input virus inoculum was pelleted through 20% sucrose and analyzed by western blotting using antibodies against CA (p30) and IN. (TIF) [file ppat.1003214.s002.tif]

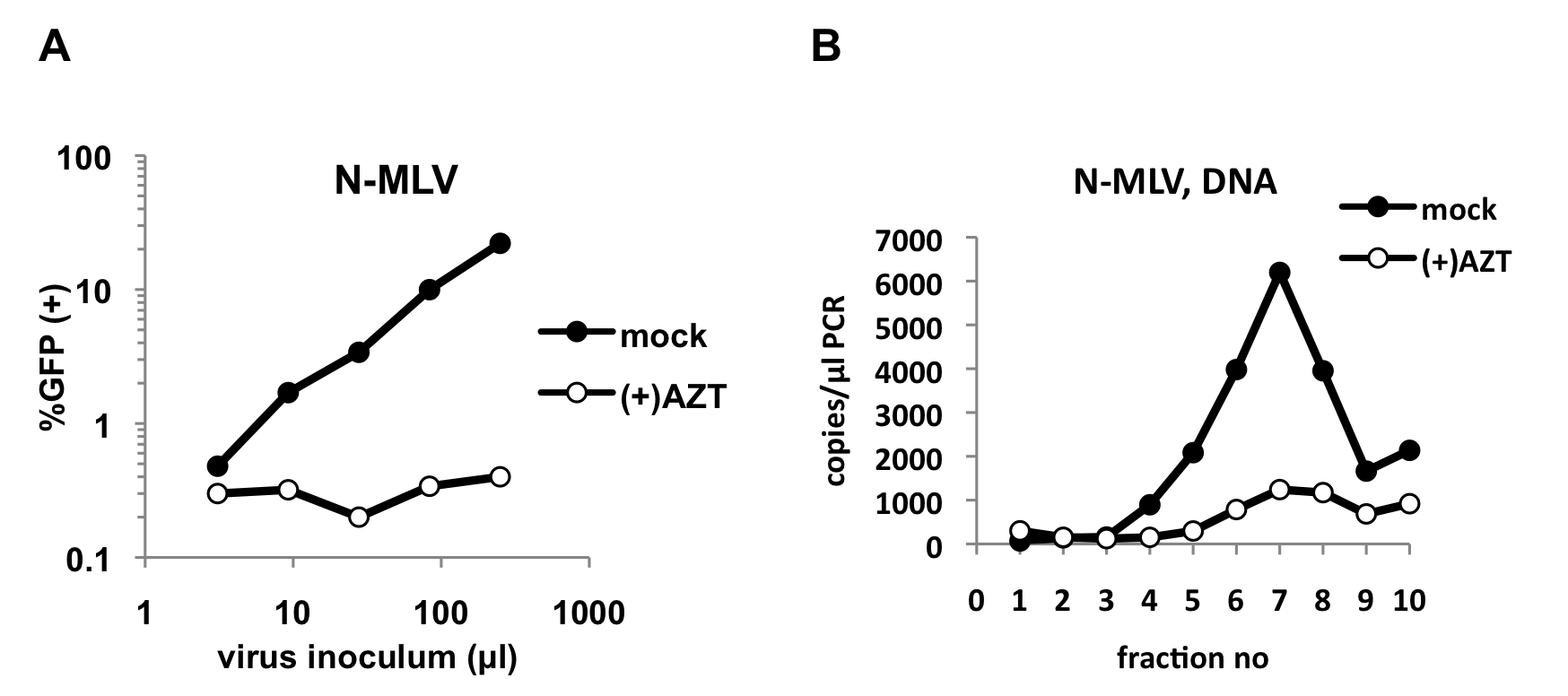

Supplement: Figure S3 — AZT blocks N-MLV infection and reverse transcription. (A, B) PgsA cells were infected with VSV-G pseudotyped N-MLV (IN-3×HA) either in the absence (mock) or in the presence of either 1 mM AZT as explained in legend to Fig. 1 and in Materials & Methods. (A) Cells were fixed and virus infectivity was determined by FACS at 2 days post infection. (B) Q-PCR analysis of reverse transcription products isolated from mock-treated and AZT-treated pgsA cells. (TIF) [file ppat.1003214.s003.tif]

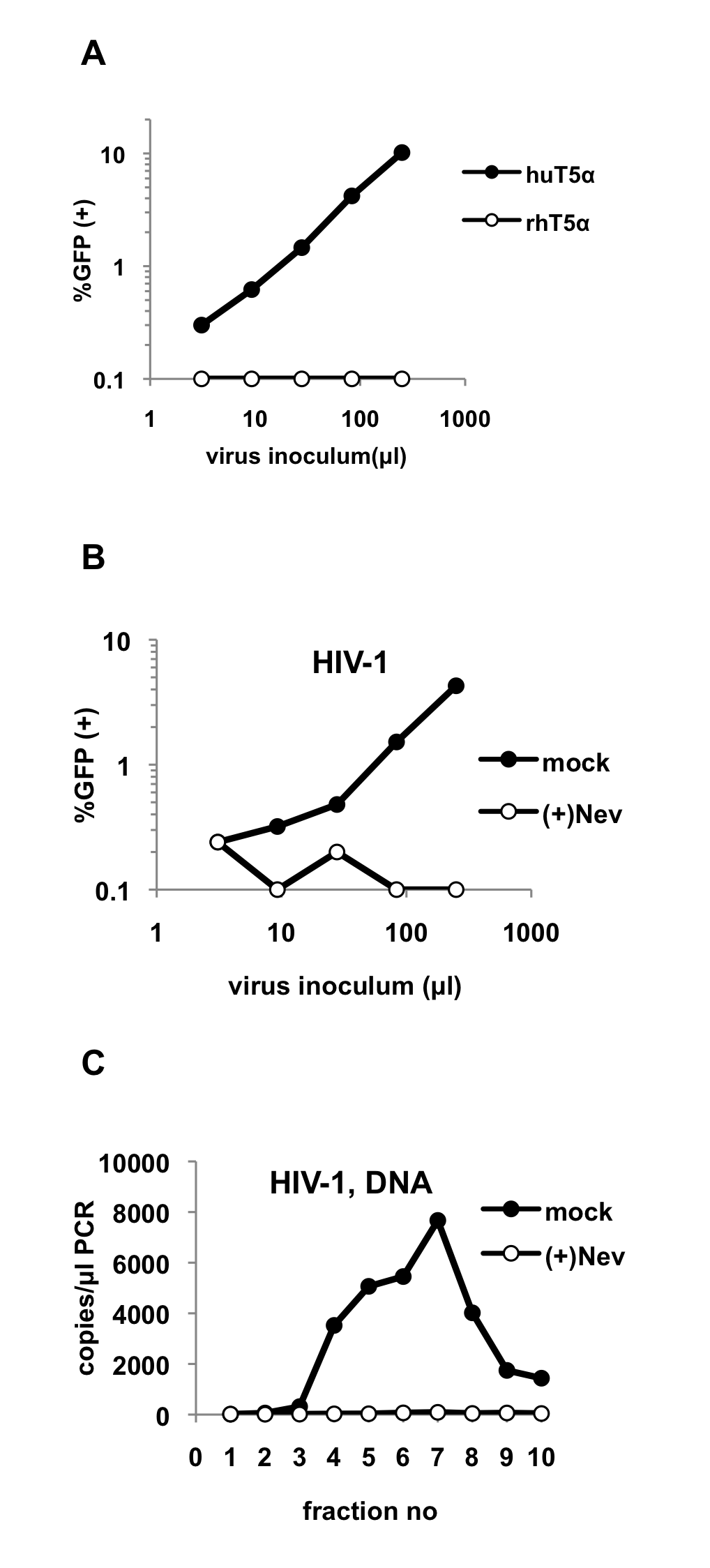

Supplement: Figure S4 — RhTRIM5α and Nevirapine block HIV-1 infection and reverse transcription. (A) PgsA-huTRIM5α (huT5α) and pgsA-rhTRIM5α (rhT5α) cells were infected with VSV-G pseudotyped HIV-1 as above and infectious titer was determined by FACS at 2 days post infection. (B, C) PgsA-huTRIM5α cells were infected by VSV-G pseudotyped HIV-1 either in the absence (mock) or in the presence of either 25 µM nevirapine as explained in legend to Fig. 1 and in Materials & Methods. (B) Cells were fixed and virus infectivity was determined by FACS at 2 days post infection. (C) Q-PCR analysis of reverse transcription products isolated from mock-treated and AZT-treated pgsA cells at 2 h post infection. (TIF) [file ppat.1003214.s004.tif]

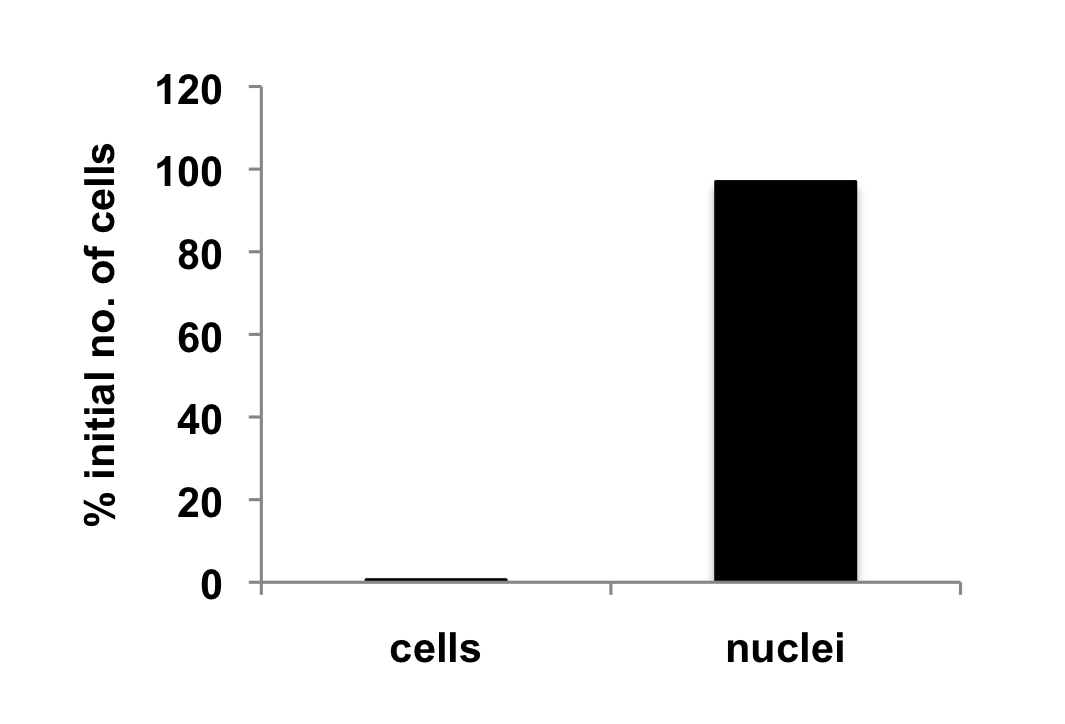

Supplement: Figure S5 — Dounce homogenization of pgsA cells. PgsA cells that were processed the same way as for the infected samples were dounce homogenized in hypotonic buffer as explained in Materials & Methods. Cell integrity before homogenization (in hypotonic buffer) and after 50 strokes was determined by Trypan blue staining of cells, and using a Countess automated cell counter. Integrity of nuclei after 50 strokes was determined by counting Trypan blue stained nuclei. Data is presented as the percentage of the initial number of cells. (TIF) [file ppat.1003214.s005.tif]
